# In silico evaluation of potential penicillin-binding protein 2 (PBP2) inhibitors from *Borassus flabellifer* haustorium against cephalosporin-resistant *Neisseria gonorrhoeae*

**DOI:** 10.3389/fphar.2026.1918221

**Published:** 2026-10-09

**Authors:** Udhaya Rajasekaran, T. B. Sridharan

**Affiliations:** Department of Integrative Biology, School of Bio Sciences and Technology, Vellore Institute of Technology, Vellore, Tamil Nadu, India

**Keywords:** antimicrobial resistance, Borassus flabellifer haustorium, molecular dynamics simulation, *Neisseria gonorrhoeae*, penicillin-binding protein 2 (PBP2)

## Abstract

**Background:**

The emergence of cephalosporin-resistant *Neisseria gonorrhoeae* strains, encoding altered Penicillin-Binding Protein 2 (PBP2), has become a major global public health concern. The increasing prevalence of antimicrobial resistance necessitates the development of novel antimicrobial agents. In the present study, phytocompounds derived from the haustorium of *Borassus flabellifer* L. were investigated as potential inhibitors of PBP2 using an integrated *in silico* approach.

**Methodology:**

Phytochemical profiling of the methanol, ethyl acetate and hexane extracts of white spongy palmyra haustorium was performed using GC–MS and the identified compounds were subsequently evaluated for molecular docking, ADMET analysis and molecular dynamics simulation.

**Results:**

A total of 69 compounds were identified and subjected to molecular docking against the transpeptidase domain of PBP2. The docking analysis revealed predicted binding affinities, ranging from −4.5 to −9.7 kcal/mol. The top ten compounds were subsequently evaluated for their ADMET and drug-likeness properties. Among these, 5-(4-nitrophenyl)-1,3,4-oxadiazol-2(5H)-one and 1,2,4-triazol-3-amine, 5-(1,3,5-trimethyl-4-pyrazolyl)amino, demonstrated comparatively favourable pharmacokinetic properties and balanced predicted ADMET profile. Furthermore, the stability of the complexes was assessed for a time period of 100 ns using molecular dynamics simulation. RMSD, RMSF, Rg, SASA, and hydrogen bonds were examined to evaluate protein-ligand interactions throughout the time frame. Principal component analysis and free energy landscape studies further supported ligand-induced stabilization of the PBP2 structure. MM-PBSA revealed favourable interaction energies of −19.11 ± 2.29 kJ/mol and −11.16 ± 2.34 kJ/mol for the two selected compounds, respectively. Among them, 5-(4-nitrophenyl)-1,3,4-oxadiazol-2(5H)-one demonstrated comparatively greater binding stability and thermodynamic favourability.

**Conclusion:**

These findings suggest that 5-(4-nitrophenyl)-1,3,4-oxadiazol-2(5H)-one could serve as a promising lead molecule for the development of new therapeutics against drug-resistant gonococcal infections and necessitate further experimental validation.

## 1 Introduction

Gonorrhoea is considered the second most common bacterial sexually transmitted infection in the world, which raises significant public health concerns along with substantial socioeconomic impact (Unemo and Shafer, 2011). The etiological agent, *N. gonorrhoeae*, is a coffee bean-shaped, Gram-negative intracellular diplococcus that primarily infects the rectum, conjunctiva, throat and mucosal surfaces of the urogenital tract. Persistent or untreated infections are associated with serious clinical outcomes, including infertility, pelvic inflammatory disease, maternal death, ectopic pregnancy, spontaneous abortion in the first trimester, adverse neonatal outcomes, and fivefold increased susceptibility to human immunodeficiency virus transmission (Yeshanew and Geremew, 2018). In 2020, the World Health Organization reported that about 82.4 million new gonorrhoea cases were globally reported within the age group of 15–49 years old. The global occurrence rate was estimated to be 23 (10–43) per 1,000 men and 19 (11–29) per 1,000 women, respectively (WHO Progress Report, 2021). An increase in gonorrhoea cases from the beginning of 2022, with notable rises among young heterosexual populations was reported by the European Centre for Disease Prevention and Control. A major determinant of gonorrhoea persistence is the high prevalence of asymptomatic infections, particularly among women, which requires timely clinical detection and diagnosis, thereby facilitating ongoing transmission (Ecdc, 2022). Due to the lack of an effective vaccine and reliable medications, disease control depends almost entirely on antimicrobial therapy (Lyu et al., 2024). However, global dissemination of antimicrobial resistance has progressively compromised the clinical treatment of gonorrhoea. *Neisseria gonorrhoeae* has developed resistance to all major antibiotic classes previously used for therapy, including sulfonamides, penicillin, tetracyclines, macrolides, fluoroquinolones, and narrow-spectrum cephalosporins (Unemo and Shafer, 2014). Extended-spectrum cephalosporin resistance (ESCR) was first identified in 2009 with the discovery of the H041 strain in Kyoto, Japan (Ohnishi et al., 2011; Shimuta et al., 2013). H041 was the first reported *N. gonorrhoeae* isolate to show high-level resistance to both cefixime and ceftriaxone (MIC = 8 μg/mL; 2–4 μg/mL), which is approximately 2000–4000-fold greater than that of wild-type strain FA19 (MIC = 0.001 μg/mL), signalling the emergence of extensively drug-resistant gonorrhoea and raising global concerns about the possibility of untreatable infections (Unemo et al., 2012). The resistance phenotype of H041 is primarily attributed to a mosaic *penA* allele which encodes a highly mutated penicillin-binding protein 2 (PBP2) containing approximately sixty amino acid changes compared to the wild-type strain (FA19) (Mlynarczyk-Bonikowska et al., 2022). The mosaic PBP2 encoded by the resistant penA allele is a major contributor to reduced susceptibility to extended-spectrum cephalosporins. Accordingly, the altered PBP2 of H041 represents a relevant target for structure-based screening of compounds against cephalosporin-resistant *N. gonorrhoeae*. Subsequently, detection of related resistant strains, including F89 in 2010 in France and Spain, and A8806 in Australia, by the end of 2013, was associated with treatment failures involving the last-line combination of ceftriaxone with azithromycin, which reinforced the urgency of this threat (Lin et al., 2021). However, declining susceptibility and documented treatment failures associated with these agents have raised serious concerns regarding the long-term sustainability of existing treatment guidelines, underscoring the urgent need to investigate and develop new antimicrobials for future therapeutic strategies.

Natural products remain a rich source of diverse bioactive compounds, especially those obtained from underexplored ecological niches. *Borassus flabellifer L. (family Arecaceae)*, commonly known as the palmyra palm, is a multipurpose perennial tropical plant widely distributed across South and Southeastern parts of Asia, viz India, Sri Lanka, Myanmar, Thailand, and Cambodia (Sathanya et al., 2024). The species is well adapted to arid and semi-arid environments and it is recognized for its longevity, robust physiology, and diverse utility in traditional food and medicinal practices (Basava Prasad et al., 2022). Several parts of the plant, including leaves, roots, seeds, and fruits, were utilized by people and are rich in glycosides, flavonoids, phenols, and saponins (Arunachalam et al., 2011). A previous study reported that phytocompounds present in its extracts possess valuable pharmaceutical properties, including antioxidant, antidiabetic, anti-inflammatory, and antimicrobial activities (Basava Prasad et al., 2021). Most phytochemical studies on *B. flabellifer* have focused on mature plant parts, leaving developmentally specialized tissues largely unexplored. The haustorium, a delicious white, spongy, edible transient tissue formed during seed germination, serves as a nutrient-absorbing interface between the endosperm and the developing embryo to support early seedling growth (Vijayakumari et al., 2017). This process involves intense enzymatic activity and biochemical reprogramming, conditions that favour the generation and accumulation of unique low molecular weight metabolites not typically observed in differentiated parts of the plant. A previous study indicates that the haustorium contains carbohydrates, proteins, minerals, and phenolic constituents (Arunachalam et al., 2011). Although the *Borassus flabellifer* haustorium has been reported to contain diverse nutritional and phytochemical constituents, its potential as a source of compounds against *N. gonorrhoeae*, particularly antimicrobial-resistant strains, remains poorly explored. Moreover, the potential of haustorium-derived phytochemicals to target the altered PBP2 of the cephalosporin-resistant H041 strain remains unexplored. Therefore, in the present study, we characterized the phytochemical constituents of *B. flabellifer* haustorium using gas chromatography–mass spectrometry (GC–MS) with solvents of differing polarity to ensure comprehensive extraction of phytochemicals across a wide polarity range and employed an integrated *in silico* approach to evaluate their interaction potential with PBP2 of the cephalosporin-resistant *N. gonorrhoeae* H041 strain using molecular docking and molecular dynamics simulations. This work aims to prioritize haustorium derived compounds for future structure guided investigation against resistant gonorrhoea.

## 2 Materials and methods

### 2.1 Sample collection

Germinated seeds of palmyra palm (*B. flabellifer* L.) were collected from agricultural farmland in Pichanandal village, Tiruvannamalai district (12.3163393, 79.0554158), Tamil Nadu. The plant material was taxonomically identified and authenticated by Dr. Rajasekaran C, Professor Grade I, Department of Biotechnology, School of Bio Sciences and Technology (SBST), VIT, Vellore, under authentication No. VITCS14. The collected seeds were transported to Gene cloning technology laboratory, VIT, Vellore, and processed immediately in order to obtain white spongy palmyra haustorium (WSPH). Furthermore, WSPH was cut into small pieces, washed thoroughly twice using distilled water, and dried at 50 °C for 24 h in a laboratory drying oven. Dried WSPH was subsequently ground into a powder, passed through an 80-mesh sieve, and stored at −20 °C for further extraction (Basava Prasad et al., 2022).

### 2.2 Extraction of WSPH

The WSPH extraction was carried out following the method described by Dadi et al. (2019) with slight modifications. Briefly, powdered WSPH was suspended separately in methanol, ethyl acetate, and hexane at a 1:10 ratio (w/v). Extraction was performed in an ultrasonic bath (Model LMUC-3, Make LAB MAN) with a constant frequency of 40 k*Hz* and power supply of AC 220 V/50 *Hz* for 30 min at solvent-specific temperatures (methanol: 64 °C; ethyl acetate: 77 °C; hexane: 68 °C). The extracts were centrifuged for 10 min at 2,100×g with an Eppendorf 5810 R centrifuge, and the collected supernatants were kept in a rotary evaporator to remove residual solvents from the samples.

### 2.3 GC–MS analysis

To characterize the bioactive compounds present in three extracts, we utilized GC–MS analysis using an Agilent GC-7890 A coupled with MS-5975C system. For chromatographic separation, a DB-5MS fused silica capillary column with an internal diameter of 30 m × 0.25 mm and 0.25 μm film thickness was utilized. An optimum flow rate (1.0 mL/min) was maintained with helium gas as carrier. The injector temperature was maintained at 250 °C, and a 1 µL aliquot of each extract was injected at a ratio of 1:10 in split mode. An initial oven temperature was maintained at 50 °C for 1 min, followed by a programmed increase of 10 °C min^-1^–300 °C with a final hold of 4 min. The temperatures of the ion source and transfer line were maintained at 230 °C and 280 °C. Electron ionization at 70 eV was utilized for mass spectral acquisition. Compound identification was carried out by comparing retention times and mass spectral fragmentation patterns with entries in the National Institute of Standards and Technology (NIST) mass spectral library (Irawan et al., 2018; Banni and Jayaraj, 2023; Metouekel et al., 2024).

### 2.4 Retrieval of lead molecules

The GC–MS identified phytocompounds of methanolic, ethyl acetate, and hexane extracts of WSPH were searched on the PubChem database (https://pubchem.ncbi.nlm.nih.gov/) (Kim et al., 2025) and their corresponding 2D and 3D conformers were retrieved in Structure Data File (SDF) format. The obtained SDF format ligand structures were processed and converted to PDBQT format using Open Babel tool (v3.1.1) for the further molecular docking studies (O’Boyle et al., 2011).

### 2.5 Preparation of receptor protein

The crystal structure of the transpeptidase domain of PBP2 from the cephalosporin-resistant *N. gonorrhoeae* H041 strain (PDB ID: 8VEN), comprising 330 amino acid residues and resolved at 1.80 Å, was obtained from Protein Data Bank (PDB) (https://www.rcsb.org/structure/8VEN) (Berman et al., 2000). Protein preparation was performed using AutoDock Tools v1.5.7. It involved removal of H_2_O molecules, ligands that are co-crystallized and non-essential ions. The receptor structural integrity was ensured by the addition of polar hydrogen atoms. Missing atoms were identified and repaired, and atomic charges were assigned with the appropriate partial charges (Gasteiger and Kollman). The prepared receptor structure was exported in PDBQT format for docking analysis (Trott and Olson, 2010).

### 2.6 Molecular docking

AutoDock Vina was used to predict the binding affinities and binding poses of the ligands toward the PBP2 active site. For comparative evaluation, ceftriaxone was included as the standard drug alongside the identified plant compounds. The active site residues were GLU307, SER310, SER362, ASN364, TYR422, SER483, THR498, THR500, ARG502, TYR509, THR517, SER545. These residues encompass the catalytic and substrate-binding domains (Turner et al., 2024). A grid box encompassing the transpeptidase active site was generated to ensure adequate conformational sampling during the docking process. The docking was performed with an 80 × 80 × 80 Å grid centred at different axes with x = −8.842, y = −3.019, and z = 16.677. The default AutoDock Vina parameters were utilized to analyse molecular docking calculations, with 10 output binding poses generated per ligand. The docking protocol was validated by redocking the crystallographic ligand from PBP2 using the same docking parameters. The crystallographic and redocked ligand poses were superimposed in PyMOL (v3.1.3) and RMSD was calculated for the 37 corresponding ligand atoms. Discovery Studio Visualizer (v24.1.0.23298) was used for performing the analysis and visualization of interactions between protein and ligands. 3D interaction models were used to examine ligand positioning within the binding pocket, while 2D interaction diagrams were produced to identify hydrophobic interactions, hydrogen bonds, and other non-covalent contacts between the ligand and active-site residues (BIOVIA, Dassault Systèmes, 2024) (Eberhardt et al., 2021).

### 2.7 ADMET and drug-likeness evaluation

The *in silico* pharmacokinetic profiling for the most potent docked phytochemicals was performed using the SwissADME web server (http://www.swissadme.ch). The obtained ligand canonical SMILES representations from the PubChem database (https://pubchem.ncbi.nlm.nih.gov/) were considered as inputs in the analysis (Daina et al., 2017). Toxicity properties were predicted using the ProTox-3.0 web server (https://tox.charite.de/) (Banerjee et al., 2024). The predicted pharmacokinetic properties were used as a preliminary filtering criterion to prioritize compounds for further molecular dynamics simulation analysis.

### 2.8 Molecular dynamics simulation

To analyse the structural dynamics of protein-ligand complexes and apo form of PBP2, molecular dynamics (MD) simulations were performed by using GROMACS 2023.1 with CHARMM force field. MD simulations were conducted under physiologically representative three-dimensional conditions to assess the flexibility of the complexes. Furthermore, GROMACS tools along with SwissParam were used to generate the topology files. The systems were solvated with TIP3P water model and appropriate counterions (Na^+^ and Cl^−^) were added to maintain the ionic strength relative to the physiological environment for stable equilibration. The systems were energy minimized through 50,000 steps of the steepest descent algorithm for each MD simulation (Huang and Mackerell, 2013; Lu et al., 2014; Waterhouse et al., 2018). Subsequently, to achieve thermodynamic stability, the system was set for a two-stage equilibration process, consisting of an NVT ensemble to regulate temperature at 300 K, followed by an NPT ensemble to stabilize pressure at 1 bar for a 100 ps interval. Temperature and pressure were regulated using the Berendsen thermostat and Parrinello–Rahman barostat, respectively (Parrinello and Rahman, 1981; Berendsen et al., 1995). The molecular dynamics production simulations were run at a timestep of 2 femtoseconds (fs) for 100 ns (Pal et al., 2023). The generated trajectories were then analysed using the built-in GROMACS utilities. Structural stability was evaluated by RMSD (root mean square deviation) values using *gmx_rms*. Residual flexibility was quantified by root mean square fluctuation (RMSF) values using *gmx_rmsf*. Structural compactness was evaluated by calculating radius of gyration (Rg) values using *gmx_gyrate*. The solvent-accessible surface area (SASA) was calculated to analyse the solvent exposure of the system using *gmx_sasa*. Protein-ligand hydrogen bond interactions throughout the simulation trajectory were analysed using *gmx_hbond*. Finally, graphical analyses were generated using QtGrace software to compare the dynamic behaviour of the apo and complex forms of protein over the simulation period (Devi and Jayaraman, 2025; Sowmeya and Sathiavelu, 2026).

### 2.9 Principal component analysis (PCA) and free energy landscape (FEL) analysis

PCA was employed to characterize the major conformational dynamics and concerted movements of the proteins and the ligand-bound complexes throughout the simulation trajectories. GROMACS analysis utilities were used to perform PCA. Initially, all trajectory frames were fitted to the reference structure using gmx_trjconv to remove overall translational and rotational movements. The gmx_covar utility was employed to calculate the covariance matrix representing the positional fluctuations of atoms. Diagonalization of this matrix yielded eigenvalues and eigenvectors describing the amplitude and direction of collective atomic motions. The essential dynamics of the system are captured by principal components obtained from eigenvectors associated with the largest eigenvalues. MD trajectories were projected onto the first two principal components of PC1 and PC2, which was performed using gmx_anaeig to visualize the dominant conformational transitions of the apo and ligand-bound complexes (Yasuda et al., 2020; Zhang et al., 2026).

To further explore the conformational space sampling and evaluate thermodynamic stability, a free energy landscape (FEL) was constructed using *gmx_sham* module in GROMACS. The FEL was generated based on the probability distribution of structures along PC1 and PC2, allowing identification of energetically favourable conformational states and transitions throughout the simulation trajectory. The Boltzmann equation was used to calculate FEL:

$$\Delta G=-K_{B\;}T\ln\left(P\right)$$

Where ΔG represents free energy, K_B_ represents the Boltzmann constant, T represents temperature, and P represents the probability density. OriginPro software was used to plot and visualize the resulting FEL data (Parida et al., 2021).

### 2.10 Binding free energy calculation

The binding free energy was calculated using the Molecular Mechanics and Poisson-Boltzmann Surface Area (MM-PBSA) approach with the *gmx_mmpbsa* tool for last 10 ns of the simulation run, which is incorporated in the GROMACS package. This approach provides insight about the thermodynamic favourability of protein-ligand interactions, integrating contributions from solvation energy, Van der Waals forces, and electrostatic interactions.

The following equation was used to calculate the total binding free energy (ΔG _bind_)

ΔG bind=ΔG complex– (ΔG protein+ΔG)ligand

ΔG _complex_ - free energy of complex, ΔG _ligand_ – ligand, ΔG _protein_ - apo protein

The more negative ΔG _bind_ values indicate an energetically favourable interaction, which implies the stability of the complex (Dong et al., 2021).

## 3 Results and discussion

### 3.1 GC–MS analysis

The WSPH extracts obtained using methanol, ethyl acetate, and hexane showed extraction yields of 1.68%, 1.24%, and 0.72%, respectively, and were analysed using GC–MS for the identification of phytochemical compounds. Figures 1–3 present the total ion chromatograms of each extract, and the identified phytocompounds are listed in Tables 1–3 according to their retention times. Extraction with solvents of varying polarity recovered different phytochemical profiles. Among the analysed extracts, the methanolic extract showed the highest number of compounds (n = 29), followed by the ethyl acetate extract (n = 28) and the hexane extract (n = 12). The methanolic extract presented the most chemical diversity, including aldehydes, organic acids, phytosterols, nitrogen-containing heterocycles, fatty acid esters and sugar derivatives (Table 1). The major compounds in the chromatographic profile were 5-hydroxymethylfurfural (31.24%), 10(E),12(Z)-conjugated linoleic acid (14.08%) and n-Hexadecanoic acid (8.00%). The presence of 5-hydroxymethylfurfural is consistent with previous studies on *B. flabellifer* (Duraipandiyan et al., 2020; Jayashri et al., 2025), where it was reported at 19.45% in the methanolic extract of the haustorium and 47.56% in the fruit extract. 5-Hydroxymethylfurfural (5-HMF) was reported to be a bioactive furan derivative with antioxidant, antibacterial, and anti-inflammatory properties (Zhao et al., 2013). 10(E),12(Z)-Conjugated linoleic acid is a conjugated fatty acid that has been reported in natural sources with antioxidant properties (Sakamoto et al., 2022; Taha et al., 2026). A number of nitrogen rich heterocyclic compounds, including triazine-, triazole-, and oxazolidinone-based structures, were also found at lower relative abundances in the WSPH methanolic extract. The ethyl acetate extract was predominantly enriched with lipid-associated compounds, including fatty acids, long-chain hydrocarbons, esters, and aromatic derivatives (Table 2). The major components included n-Hexadecanoic acid (27.58%), oleic acid (22.67%), and octadecanoic acid (11.26%), accounting for a substantial proportion of the total chromatographic area. These fatty acids have also been reported in a previous study of *B. flabellifer* haustorium as palmitic acid, oleic acid, and stearic acid. n-Hexadecanoic acid and octadecanoic acid show anti-inflammatory and antimicrobial properties against both Gram-positive and Gram-negative bacteria, while oleic acid has also been associated with various biological activities (Narayanankutty et al., 2023; Luz et al., 2025). The hexane extract showed a simpler, nonpolar profile characterized by aromatic esters and long-chain fatty acids. The most abundant constituents were tris(2,4-di-ter-butylphenyl) phosphate (35.47%) and phthalic acid, di (2-propylpentyl) ester (24.53%), followed by 6-octadecenoic acid (12.14%), floridanine (7.71%), and n-Hexadecanoic acid (7.17%) (Table 3). In addition, structurally distinct heterocyclic compounds such as 5-(4-nitrophenyl)-1,3,4-oxadiazol-2(5H)-one was detected at lower abundance. Overall, GC–MS profiling demonstrated that methanol extracted the broadest range of chemically diverse phytocompounds, whereas ethyl acetate and hexane preferentially enriched lipid-dominated and nonpolar compounds, respectively. Based on these findings, the phytocompounds identified by GC–MS were further considered for subsequent structure based *in silico* analysis against the selected protein target.

**FIGURE 1:**
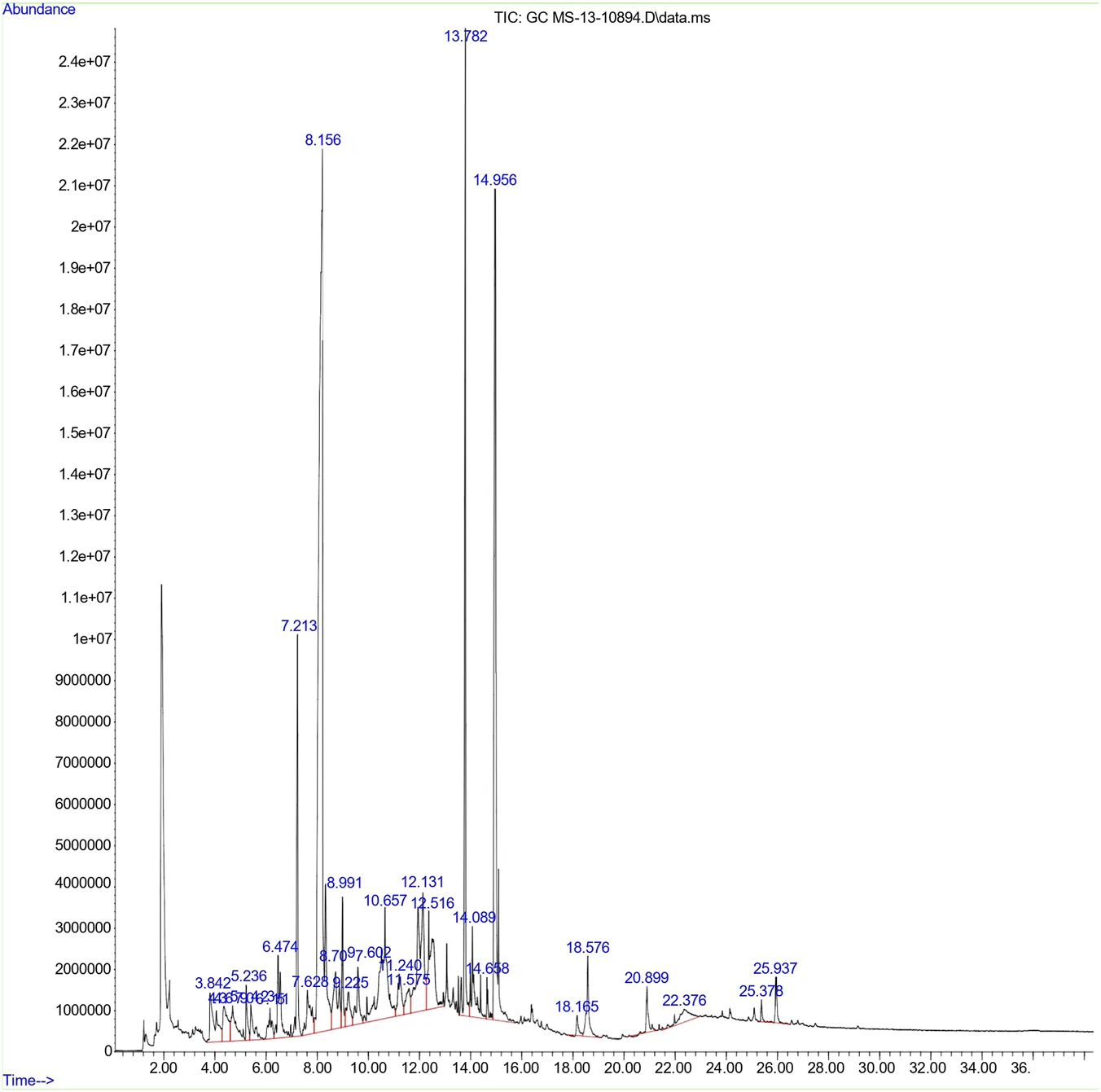
GC–MS chromatogram of methanolic extract of WSPH.

**FIGURE 2:**
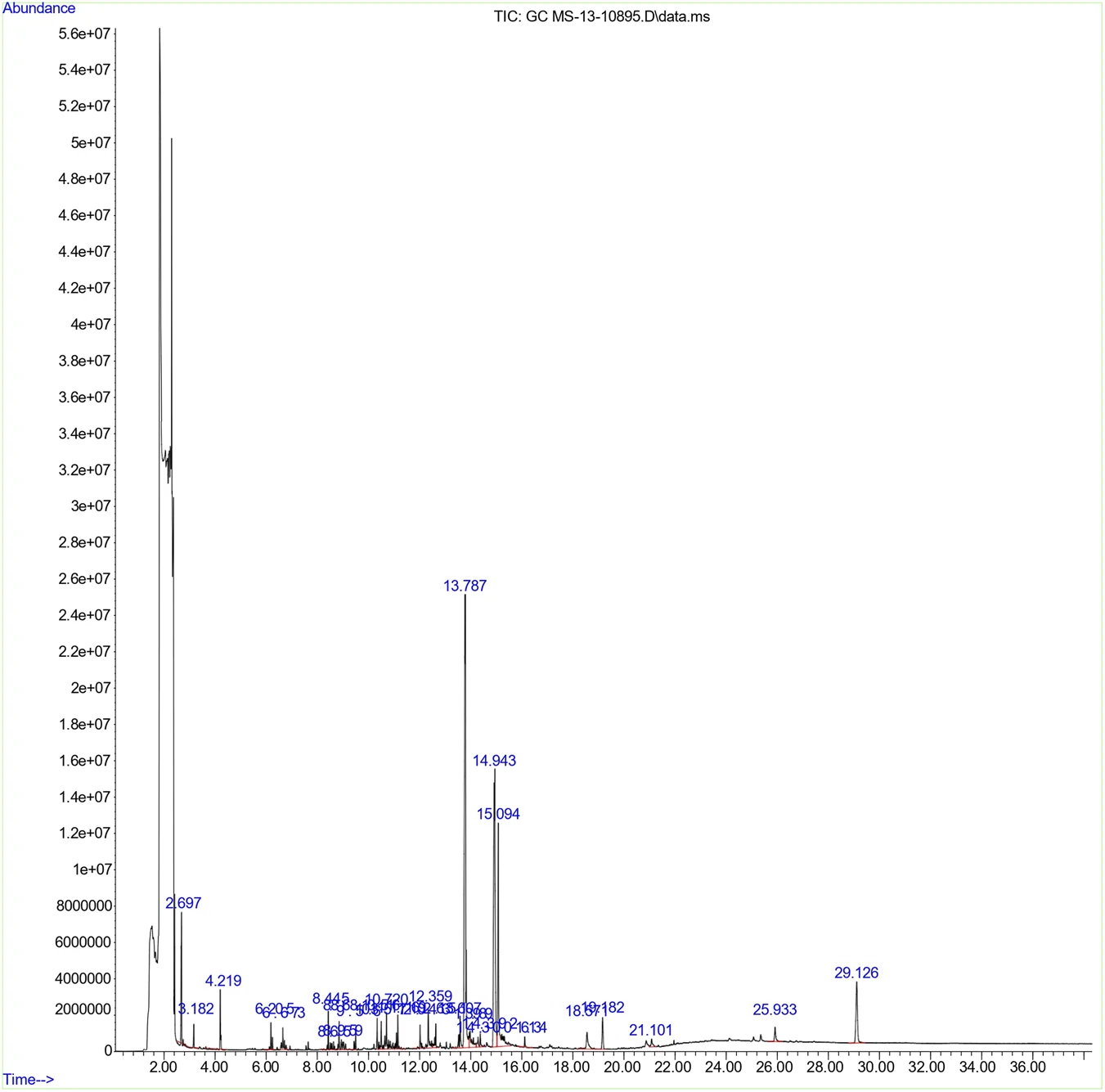
GC–MS chromatogram of ethyl acetate extract of WSPH.

**FIGURE 3:**
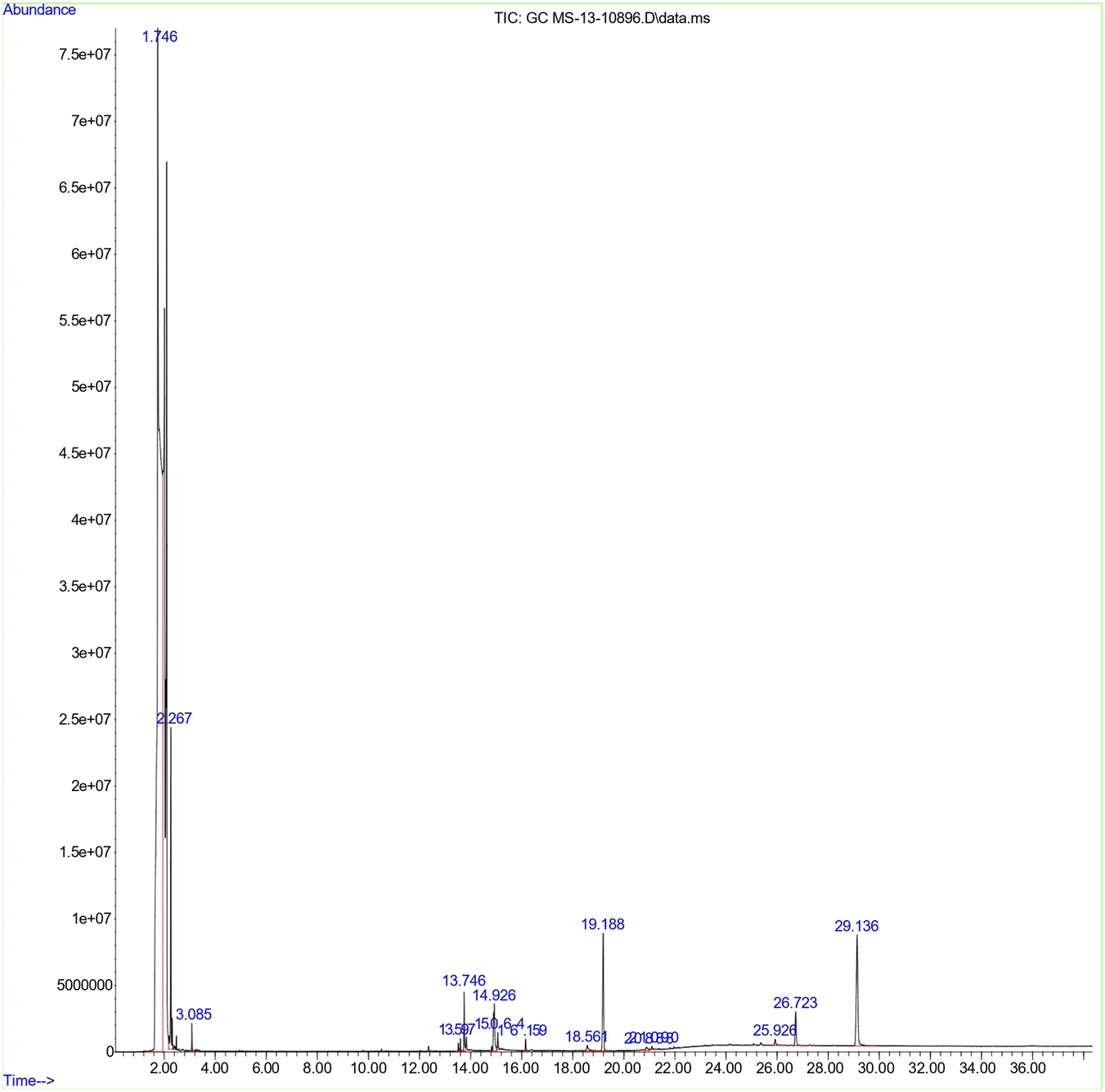
GC–MS chromatogram of hexane extract of WSPH.

**TABLE 1:** Compounds identified in methanolic extract of WSPH and their molecular interaction binding scores with targeted protein.

Peak	CID	Compound name	Structure	Retention time (min)	Molecular weight (g/mol)	Molecular formula	Peak area	Peak area %	Binding score (kcal/mol)
1	7362	Furfural	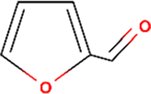	3.808	96.08	C_5_H_4_O_2_	161833600	2.07	−4.9
2	568339	N-Hydroxy-N-ethylcarbamic acid, 2-(methoxycarbonylamino)ethyl ester	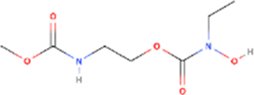	4.367	206.20	C_7_H_14_N_2_O_5_	115229242	1.47	−5.5
3	535845	Aspartic acid methyl ester	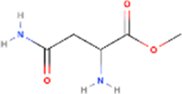	4.690	146.14	C_5_H_10_N_2_O_3_	112819023	1.44	−5.3
4	12097	2-Furancarboxaldehyde, 5-methyl	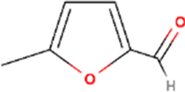	5.236	110.11	C_6_H_6_O_2_	52269962	0.67	−5.0
5	538757	2,4-Dihydroxy-2,5-dimethyl-3(2H)-furan-3-one	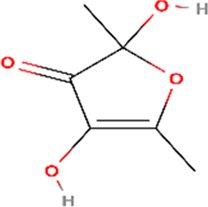	5.423	144.12	C_6_H_8_O_4_	69741417	0.89	−5.4
6	118073	Propanoic acid, 3-(acetylthio)-2-methyl-, (S)	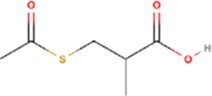	6.151	162.21	C_6_H_10_O_3_S	61725462	0.79	−5.0
7	7955	1,3,5-Triazine-2,4,6-triamine	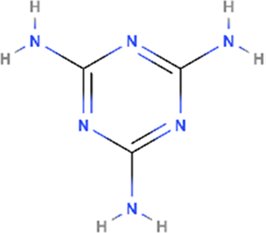	6.474	126.12	C_3_H_6_N_6_	171931732	2.20	−5.5
8	119838	4H-pyran-4-one, 2,3-dihydro-3,5-dihydroxy-6-methyl	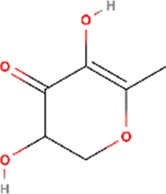	7.213	144.12	C_6_H_8_O_4_	321480821	4.11	−5.3
9	97704	3,6-Dimethylpiperazine-2,5-dione	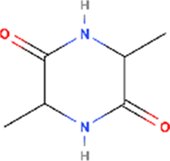	7.628	142.16	C_6_H_10_N_2_O_2_	123463008	1.58	−5.1
10	237332	5-Hydroxymethylfurfural	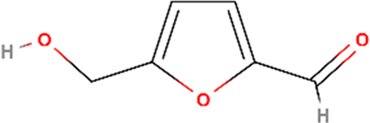	8.156	126.11	C_6_H_6_O_3_	2441510476	31.24	−5.7
11	539386	Oxazolidin-2-one, 3-(2-aminoethyl)-4-hydroxy-4-methyl-5-spiro-cyclohexane	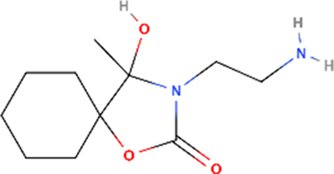	8.707	228.29	C_11_H_20_N_2_O_3_	185821455	2.38	−6.3
12	9569412	2,6-Piperazinedione, dioxime	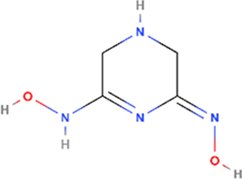	8.991	144.13	C_4_H_8_N_4_O_2_	107036528	1.37	−5.7
13	522748	Pentanoic acid, undecyl ester	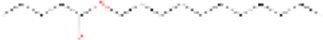	9.225	256.42	C_16_H_32_O_2_	81891208	1.05	−5.3
14	10910689	.alpha.-D-Xylofuranoside, methyl 2,3,5-tri-O-methyl	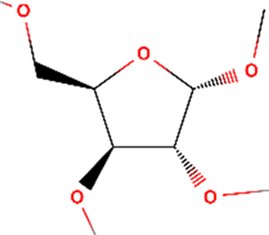	9.602	206.24	C_9_H_18_O_5_	108802799	1.39	−5.0
15	90232	Benzoic acid, 4-ethoxy-, ethyl ester	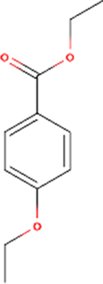	10.657	194.23	C_11_H_14_O_3_	471340769	6.03	−6.4
16	13416732	1H-1,2,3-triazolo [4,5-day]pyrimidin-7-ol, 5-mercapto	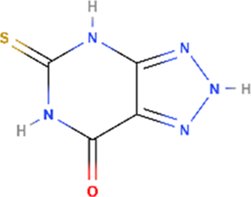	11.240	169.17	C_4_H_3_N_5_OS	97720190	1.25	−5.9
17	12889245	Methyl alpha-D-ribofuranoside	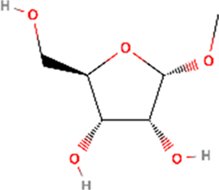	11.575	164.16	C_6_H_12_O_5_	70768908	0.91	−5.3
18	136291312	4-(Dimethylamino)-6-(methylamino)-1,3,5-triazin-2-ol	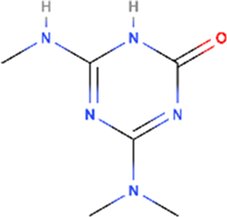	12.131	169.19	C_6_H_11_N_5_O	461375963	5.90	−5.6
19	560150	2-O-Methyl-D-mannopyranosa	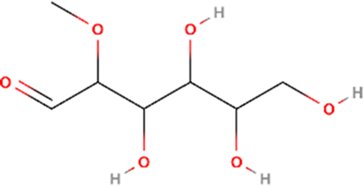	12.516	194.18	C_7_H_14_O_6_	314807076	4.03	−5.5
20	985	n-Hexadecanoic acid	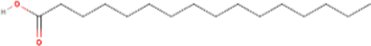	13.782	256.42	C_16_H_32_O_2_	625449762	8.00	−5.2
21	12366272	Cirsiumaldehyde	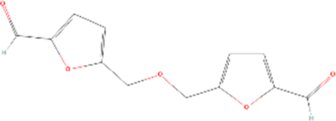	14.089	234.20	C_12_H_10_O_5_	166364310	2.13	−6.3
22	6032867	9-Octadecenoic acid, methyl ester, (E)	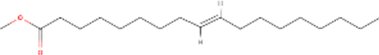	14.658	296.5	C_19_H_36_O_2_	32843495	0.42	−6.5
23	5282800	10(E),12(Z)-conjugated linoleic acid	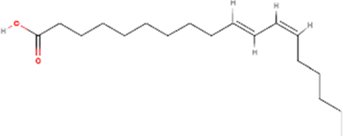	14.956	280.4	C_18_H_32_O_2_	1100259294	14.08	−5.6
24	5283321	2,4-Heptadienal, (E,E)	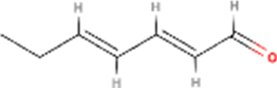	18.165	110.15	C_7_H_10_O	26001060	0.33	−4.8
25	123409	Hexadecanoic acid, 2-hydroxy-1-(hydroxymethyl)ethyl ester	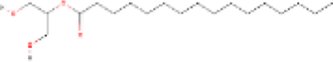	18.576	330.5	C_19_H_38_O_4_	117816597	1.51	−6.5
26	10944926	(R)-(−)-14-Methyl-8-hexadecyn-1-ol	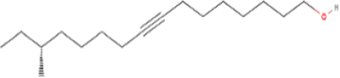	20.899	252.4	C_17_H_32_O	58874611	0.75	−5.4
27	610065	1,2,4-Triazol-3-amine, 5-(1,3,5-trimethyl-4-pyrazolyl)amino	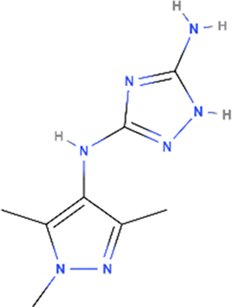	22.376	207.24	C_8_H_13_N_7_	95183749	1.22	−6.8
28	5280794	Stigmasterol	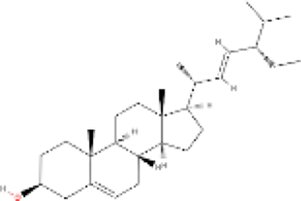	25.378	412.7	C_29_H_48_O	19497327	0.25	−9.7
29	457801	γ-sitosterol	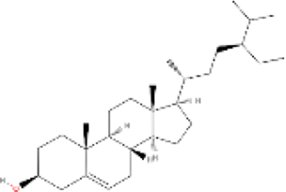	25.937	414.7	C_29_H_50_O	40823564	0.52	−9.2

**TABLE 2:** Compounds identified in ethyl acetate extract of WSPH and their molecular interaction binding scores with targeted protein.

Peak	CID	Compound name	Structure	Retention time (min)	Molecular weight (g/mol)	Molecular formula	Peak area	Peak area %	Binding score (kcal/mol)
1	31276	1-Butanol, 3-methyl-, acetate	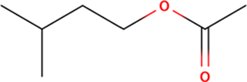	4.219	130.18	C_7_H_14_O_2_	37992103	1.33	−4.5
2	519256	Decane, 4-ethyl	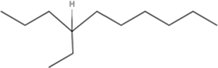	6.205	170.33	C_12_H_26_	27198974	0.95	−4.7
3	12523	Hexadecane, 2,6,10,14-tetramethyl	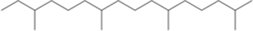	6.673	282.5	C_20_H_42_	41692056	1.46	−5.7
4	537330	Decane, 2,3,6-trimethyl	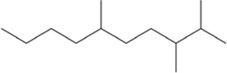	8.445	184.36	C_13_H_28_	26397369	0.93	−4.9
5	13257219	Docosane, 1-iodo	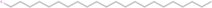	8.655	436.5	C_22_H_45_I	21711613	0.76	−6.1
6	537332	Octane, 5-ethyl-2-methyl	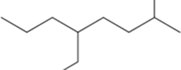	8.868	156.31	C_11_H_24_	25432142	0.89	−5.2
7	18591	Octane, 2-methyl	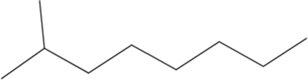	8.959	128.25	C_9_H_20_	28546382	1.00	−4.5
8	12389	Tetradecane	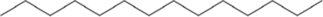	9.516	198.39	C_14_H_30_	21729016	0.76	−4.9
9	8222	Eicosane	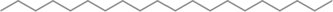	10.357	282.5	C_20_H_42_	27550116	0.97	−6.1
10	7311	2,4-Di-tert-butylphenol	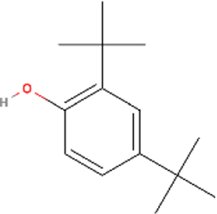	10.516	206.32	C_14_H_22_O	26370250	0.93	−6.8
11	6420421	Sulfurous acid, hexyl pentyl ester	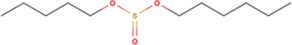	10.720	236.37	C_11_H_24_O_3_S	62359355	2.19	−5.0
12	11006	Hexadecane	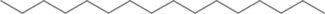	11.169	226.44	C_16_H_34_	51900756	1.82	−4.6
13	12798926	2-Bromotetradecane	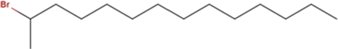	12.043	277.28	C_14_H_29_Br	26877231	0.94	−4.8
14	11005	Tetradecanoic acid	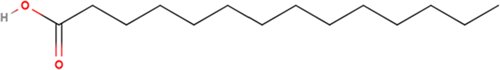	12.359	228.37	C_14_H_28_O_2_	64295205	2.26	−5.5
15	11635	Octadecane	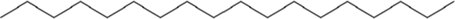	12.651	254.5	C_18_H_38_	35539665	1.25	−4.8
16	545303	7,9-Di-tert-butyl-1-oxaspiro (4,5)deca-6,9-diene-2,8-dione	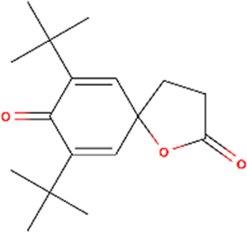	13.607	276.4	C_17_H_24_O_3_	50930501	1.79	−6.6
17	985	n-Hexadecanoic acid	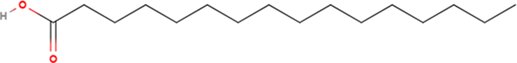	13.787	256.42	C_16_H_32_O_2_	786150768	27.58	−5.2
18	12398	Heptadecane	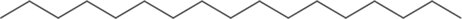	13.989	240.5	C_17_H_36_	60408359	2.12	−5.0
19	74867	3-Acetylphenanthrene	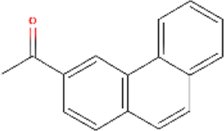	14.300	220.26	C_16_H_12_O	16234729	0.57	−9.0
20	10465	Heptadecanoic acid	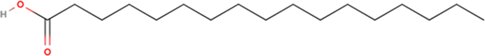	14.392	270.5	C_17_H_34_O_2_	17513004	0.61	−5.3
21	445639	Oleic acid	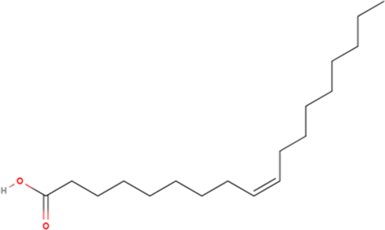	14.943	282.5	C_18_H_34_O_2_	646271356	22.67	−5.6
22	5281	Octadecanoic acid	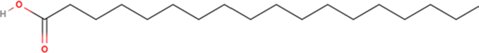	15.094	284.5	C_18_H_36_O_2_	320846686	11.26	−6.4
23	67525	Cyclopentadecane	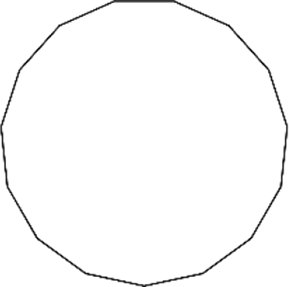	16.134	210.40	C_15_H_30_	6838747	0.24	−7.5
24	123409	Hexadecanoic acid, 2-hydroxy-1 (hydroxymethyl)ethyl ester	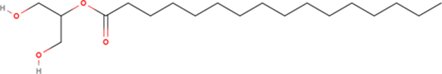	18.571	330.5	C_19_H_38_O_4_	54248894	1.90	−6.6
25	8343	Bis(2-ethylhexyl) phthalate	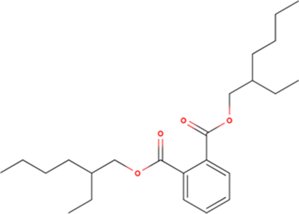	19.182	390.6	C_24_H_38_O_4_	48432873	1.70	−6.3
26	79075	Octadecanoic acid, 2-hydroxy-1-(hydroxymethyl)ethyl ester	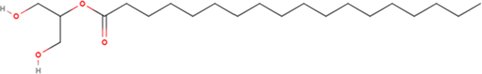	21.101	358.6	C_21_H_42_O_4_	18909791	0.66	−6.3
27	22303375	Acetic acid, 2-[bis(methylthio)methylene]-1-phenylhydrazide	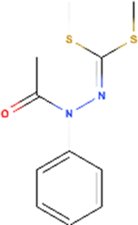	25.933	254.4	C_11_H_14_N_2_OS_2_	27851227	0.98	−6.0
28	14572930	Tris (2,4-di-tert-butylphenyl) phosphate	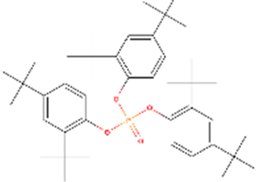	29.126	662.9	C_42_H_63_O_4_P	138696749	4.87	−7.9

**TABLE 3:** Compounds identified in hexane extract of WSPH and their molecular interaction binding scores with targeted protein.

Peak	CID	Compound name	Structure	Retention time (min)	Molecular weight (g/mol)	Molecular formula	Peak area	Peak area %	Binding score (kcal/mol)
1	545303	7,9-Di-tert-butyl-1 oxaspiro (4,5)deca-6,9-diene-2,8-dione	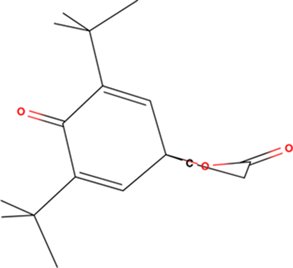	13.597	276.4	C_17_H_24_O_3_	2439081	2.33	−7.3
2	985	n-Hexadecanoic acid	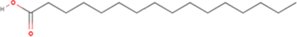	13.746	256.42	C_16_H_32_O_2_	7490218	7.17	−5.4
3	11634	6-Octadecenoic acid	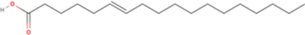	14.926	282.5	C_18_H_34_O_2_	12686903	12.14	−5.6
4	5281	Octadecanoic acid	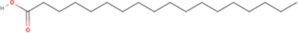	15.064	284.5	C_18_H_36_O_2_	2420388	2.32	−6.4
5	543923	Methyl 8-(3-octyl-2-oxiranyl)octanoate	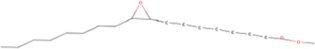	16.159	312.5	C_19_H_36_O_3_	1563223	1.50	−6.6
6	14900	Glycerol 1-palmitate	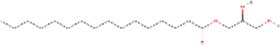	18.561	330.5	C_19_H_38_O_4_	2593591	2.48	−6.5
7	191964	Phthalic acid, di (2-propylpentyl) ester	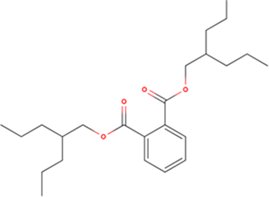	19.188	390.6	C_24_H_38_O_4_	25633203	24.53	−6.6
8	90107969	12-Methyl-e,E−2,13-octadecadien-1-ol	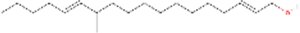	20.888	280.5	C_19_H_36_O	1910518	1.83	−5.4
9	79075	Octadecanoic acid, 2-hydroxy-1-(hydroxymethyl)ethyl ester	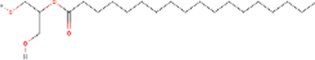	21.090	358.6	C_21_H_42_O_4_	972929	0.93	−6.1
10	610108	5-(4-nitrophenyl)-1,3,4-oxadiazol-2(5H)-one	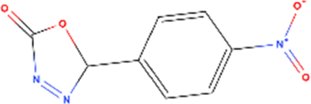	25.926	207.14	C_8_H_5_N_3_O_4_	1666729	1.60	−7.1
11	16401574	Floridanine	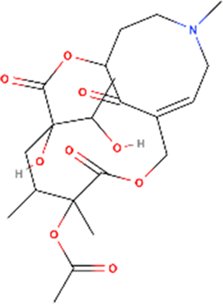	26.723	441.5	C_21_H_31_NO_9_	8055959	7.71	−7.6
12	14572930	Tris (2,4-di-tert-butylphenyl) phosphate	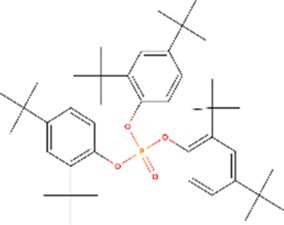	29.136	662.9	C_42_H_63_O_4_P	37064924	35.47	−8.2

### 3.2 Molecular docking analysis

Molecular docking was used to simulate the interaction of the phytocompounds from WSPH extracts with the receptor protein (PBP2). The docking protocol was validated by redocking the crystallographic ligand from PBP2, which reproduced the experimentally observed binding orientation with an RMSD of 1.588 Å across 37 corresponding ligand atoms following structural superposition, supporting the reliability of the docking protocol. Ceftriaxone was used as reference for comparative evaluation of the phytocompounds, and its predicted binding interactions with PBP2 are presented in Supplementary Figure S1. The predicted binding affinities of the phytocompounds ranged from −4.5 to −9.7 kcal/mol (Tables 1–3), with several compounds exhibiting favourable predicted interactions within the PBP2 binding pocket. Detailed hydrogen-bonding and non-covalent interactions of the ten potent compounds, which displayed lower binding energies (−6.8 to −9.7 kcal/mol), are illustrated in Table 4. The observed variation in binding energies shows differential interaction strengths among structurally diverse ligands derived from methanolic, ethyl acetate, and hexane extracts of WSPH.

**TABLE 4:** Top ten binding affinity phytocompounds of WSPH extracts and their 2D interactions with active site of targeted protein.

Compound name	Binding scores (kcal/mol)	Number of H-bond	2D interactions
Stigmasterol	−9.7	0	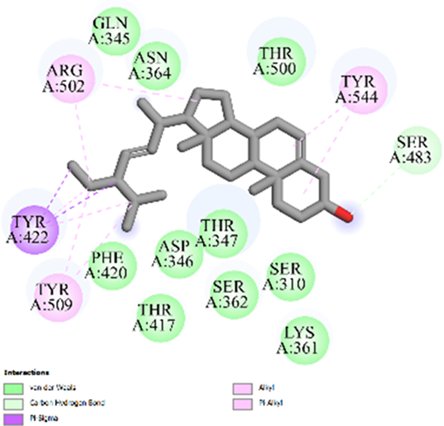
γ-sitosterol	−9.2	0	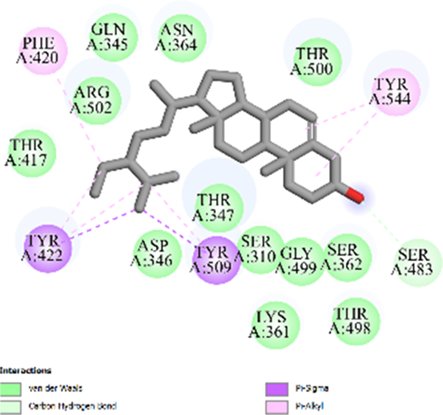
3-Acetylphenanthrene	−9.0	2 – Tyr-A:422, Arg-A:502	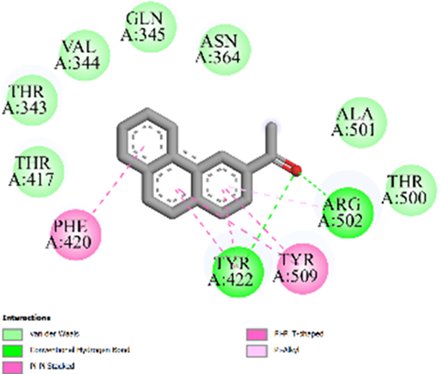
Tris (2,4-di-tert-butylphenyl) phosphate	−8.2	0	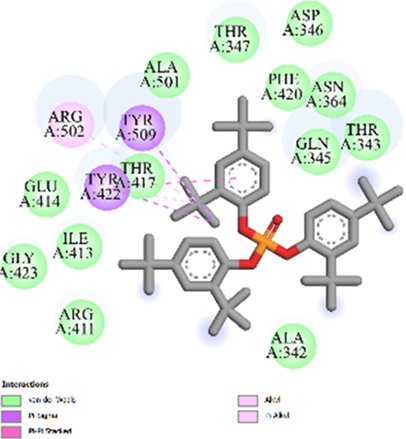
Floridanine	−7.6	2 – Thr-A:498, Thr-A:500	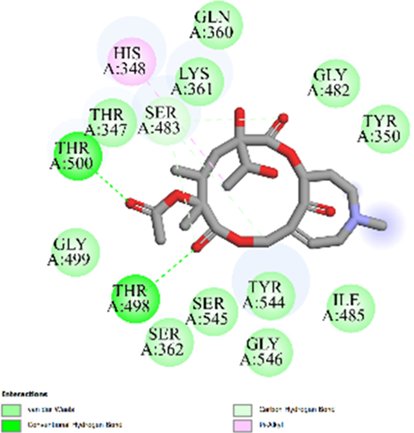
Cyclopentadecane	−7.5	0	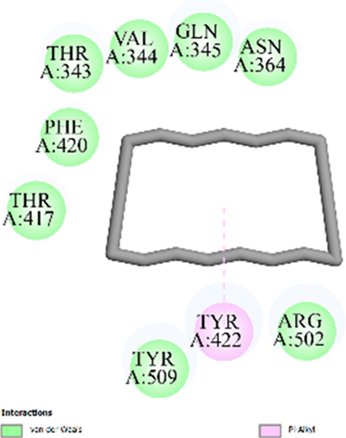
7,9-Di-tert-butyl-1 oxaspiro (4,5)deca-6,9-diene-2,8-dione	−7.3	1 – Asn-A:364	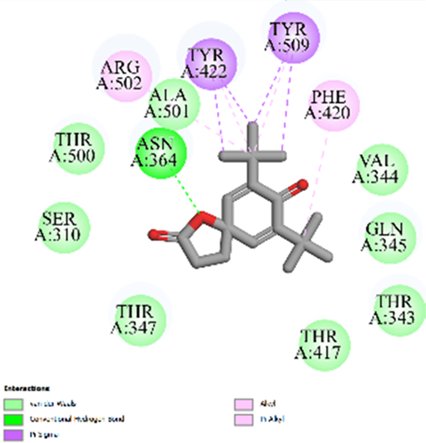
5-(4-nitrophenyl)-1,3,4-oxadiazol-2(5H)-one	−7.1	2 – Thr-A:264, Thr-A:534	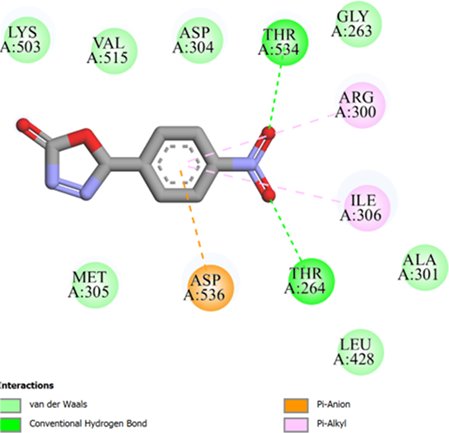
1,2,4-Triazol-3-amine, 5-(1,3,5-trimethyl-4-pyrazolyl)amino	−6.8	4 – Lys-A:313, Asn-A:364, Tyr-A:422, Thr-A:500	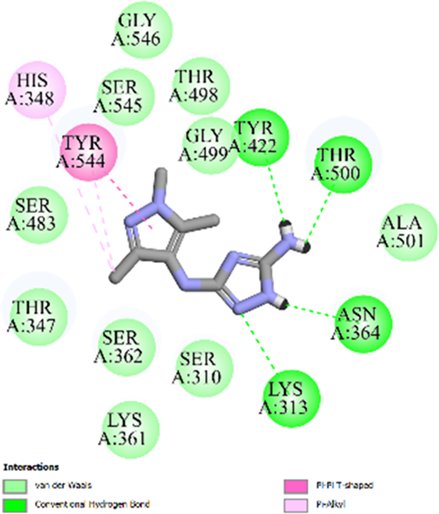
2,4-Di-tert-butylphenol	−6.8	1 – Arg-A:502	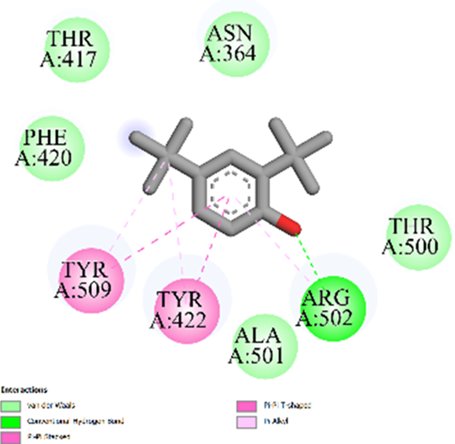

Small polar metabolites and simple heterocyclic compounds generally showed weaker binding affinities, whereas aromatic, long-chain aliphatic, and sterol-based compounds showed comparatively stronger interactions within the binding pocket of target protein. Among the top ten phytocompounds, sterol-based molecules such as stigmasterol and γ-sitosterol identified in the methanolic extract demonstrated the stronger binding affinities with −9.7 and −9.2 kcal/mol. Notably, these compounds lack conventional hydrogen bonds with the target protein; instead, their stable binding was primarily mediated by π-alkyl interactions, Van der Waals forces, and hydrophobic contacts with residues Tyr544, Tyr422, Tyr509, Phe420, Arg502, and Ser483. A similar binding pattern was observed in case of stigmasterol in previous antimicrobial docking studies, where its interaction with bacterial PBP was largely associated with hydrophobic contacts and was supported by antibacterial activity (Lestari et al., 2024). It has also been reported that stigmasterol interacts favourably with PBP2a and exhibits antibacterial activity against methicillin-resistant *Staphylococcus aureus* (MRSA) in an *in silico* and experimental study (Danta et al., 2024). 3-acetylphenanthrene was found to have a predicted binding affinity score of −9.0 kcal/mol, stabilized through multiple π–π interactions (Phe420 and Tyr509), along with two hydrogen bonds involving Tyr422 and Arg502. The involvement of Phe420 and Tyr422 is consistent with previous molecular docking studies of *N. gonorrhoeae* PBP2, in which the phenyl ring of a screened inhibitor was positioned within a shallow hydrophobic pocket formed by these residues (Fedarovich et al., 2012). Previous studies have also reported interactions of Tyr422 and Tyr509 with aromatic groups of PBP2 ligands, including an interaction between Tyr422 and the aromatic ring of ceftriaxone, supporting the relevance of these residues in ligand binding (Singh et al., 2019). The compounds tris(2,4-di-tert-butylphenyl) phosphate (−8.2 kcal/mol) and cyclopentadecane (−7.5 kcal/mol) also showed stable binding even though there were no conventional hydrogen bonds. Their docking conformations were predominantly stabilized by residues such as Arg502, Tyr509, and Tyr422 involving π-π interactions, π-alkyl, and Van der Waals forces. However, heterocyclic and moderately polar compounds, such as floridanine and 7,9-Di-tert-butyl-1-oxaspiro (4,5)deca-6,9-diene-2,8-dione, were found to have moderate binding affinities of −7.6 kcal/mol and −7.3 kcal/mol. Floridanine formed two hydrogen bonds, while 7,9-di-tert-butyl-1-oxaspiro (4,5)deca-6,9-diene-2,8-dione formed one hydrogen bond with key residues Thr498, Thr500, and Asn364. A study by (Zaidi et al., 2025) reported favourable interactions of 7,9-di-tert-butyl-1-oxaspiro (4,5)deca-6,9-diene-2,8-dione with penicillin-binding proteins and other bacterial cell-wall proteins involved in peptidoglycan biosynthesis, with a binding affinity of −6.8 kcal/mol against PBP2a, together with antibacterial activity against MRSA. Specifically, 5-(4-nitrophenyl)-1,3,4-oxadiazol-2(5H)-one showed a binding affinity of −7.1 kcal/mol and established two hydrogen bonds with Thr264 and Thr534. Although experimental evidence for this specific compound remains limited, previous studies on related 1,3,4-oxadiazole derivatives have reported antibacterial effects against both Gram-positive and Gram-negative bacteria, with some derivatives showing bactericidal activity against MRSA *in vitro*, including activity against biofilms (Zheng et al., 2018; Belkhadem et al., 2025). Further studies on oxadiazole based antibiotics have extended these findings to *in vivo* models, with ND-421 showing efficacy against MRSA and synergistic effects when combined with β-lactam antibiotics such as oxacillin, piperacillin, imipenem, meropenem and cefepime (Janardhanan et al., 2016). Whereas 1,2,4-triazol-3-amine, 5-(1,3,5-trimethyl-4-pyrazolyl)amino displayed a moderate binding affinity of −6.8 kcal/mol but formed the highest number of hydrogen bonds (four) among the top ten compounds, interacting with Lys313, Asn364, Tyr422, and Thr500. The involvement of these residues is relevant as they are located within functionally important regions of the PBP2 binding site. Lys313 and Thr500 are part of the conserved regions that contribute to ligand recognition and active-site interactions, while Tyr422 is positioned within the β3–β4 loop and also contribute to ligand stabilization within the binding site (Fenton et al., 2021). Similarly, the alkylated phenolic compound 2,4-di-tert-butylphenol demonstrated a binding affinity of −6.8 kcal/mol and formed a hydrogen bond with Arg502. The antibacterial relevance of 2,4-di-tert-butylphenol has been reported previously, including antibiofilm activity against Group A *Streptococcus* (Viszwapriya et al., 2016) and antibacterial activity against *Cutibacterium acnes* (Rouvier et al., 2024). Furthermore, studies have reported similar interactions of 2,4-di-tert-butylphenol with penicillin-binding proteins. Sundar and Arunachalam (2025) reported a binding affinity of −6.3 kcal/mol against PBP2a from MRSA, while Ali et al. (2026) reported a binding affinity of −6.2 kcal/mol against PBP2 of *P. aeruginosa*. Collectively, these interaction patterns of the compounds suggest that polar functional groups enhance specificity and anchoring within the active site, with reduced overall hydrophobic stabilization compared to sterol-like ligands. The continued involvement of residues Tyr422, Tyr509, Arg502, Thr500, Ser483, and Asn364 across the most stable complexes highlights their role as key interaction residues within the PBP2 transpeptidase domain and their importance in ligand mediated inhibition.

### 3.3 ADME and drug-likeness evaluation

The top ten low-binding-energy phytocompounds from WSPH extracts were identified through molecular docking analysis. These were subsequently evaluated for their pharmacokinetic behaviour, physicochemical properties, and drug-likeness parameters. The results are mentioned in Table 5. To assess the drug-likeness properties of the selected phytocompounds, Lipinski’s Rule of Five was used as the primary criterion. The other evaluated parameters include molar refractivity (MR), topological polar surface area (TPSA), gastrointestinal (GI) absorption, P-glycoprotein (P-gp) substrate prediction, cytochrome P450 (CYP) inhibition liability, blood-brain barrier (BBB) permeability, and aqueous solubility (Log *S*). These parameters were considered, as they collectively influence the metabolic stability, distribution behaviour, membrane permeability, and oral bioavailability of small molecules (Linker et al., 2023). The results demonstrated that out of ten phytocompounds, six fulfilled Lipinski’s rule of five without any violations, whereas three compounds, *viz.*, stigmasterol, γ-sitosterol, and cyclopentadecane, exhibited a single violation yet remained within acceptable drug-likeness limits. However, tris(2,4-di-tert-butylphenyl) phosphate showed two violations due to its high molecular weight and Log*P* values, suggesting that it may have limited suitability as an orally bioavailable drug candidate (Soni et al., 2026). Compounds violating one or more of these criteria are generally associated with poor absorption or limited membrane permeability (Benet et al., 2016). Among the selected phytocompounds nine showed molecular weights within the acceptable range, between 206.32 and 441.47 g/mol except tris(2,4-di-tert-butylphenyl) phosphate (662.92 g/mol) (Tables 1–3). The lowest molecular weights were observed for 2,4-di-tert-butylphenol (206.32 g/mol), 5-(4-nitrophenyl)-1,3,4-oxadiazol-2(5H)-one (207.14 g/mol), and 1,2,4-triazol-3-amine, 5-(1,3,5-trimethyl-4-pyrazolyl)amino (207.24 g/mol). Floridanine (10) has the highest number of hydrogen bond acceptors (HBA) compared to other phytocompounds, followed by 5-(4-nitrophenyl)-1,3,4-oxadiazol-2(5H)-one, which has 6 HBA. The maximum number (3) of hydrogen bond Donors (HBD) was found in 5-(1,3,5-trimethyl-4-pyrazolyl)amino among the selected phytocompounds. All the selected phytocompounds showed ≤10 HBA and ≤5 HBD, indicating a balanced hydrogen bonding profile, favourable for oral bioavailability (Veber et al., 2002). The presence of these HBA and HBD functionalities helps in intermolecular hydrogen bonding and improves the aqueous solubility, which are critical determinants of drug absorption (Javed et al., 2024). Four phytocompounds demonstrated a violation of Lipinski’s rule due to elevated lipophilicity, as reflected by the high Log *P* values of tris(2,4-di-tert-butylphenyl) phosphate (12.15), stigmasterol (6.98), γ-sitosterol (7.24), and cyclopentadecane (5.1), whereas the remaining six phytocompounds showed <5 Log *P* values. Higher Log *P* values (>5) indicate increased lipophilicity, which may result in prolonged retention of compound within lipid bilayers and an increased risk of toxicity (Pajouhesh and Lenz, 2026). TPSA was calculated for the selected phytocompounds as an indicator of molecular polarity and membrane permeability. In general, compounds with TPSA values between 20 and 130 Å^2^ exhibit favourable membrane permeation and are often associated with reduced adverse side effects (Lesani et al., 2025). The analysis revealed that cyclopentadecane and acetylphenanthrene exhibited very low TPSA values of 0 Å^2^ and 17.07 Å^2^ respectively, indicating low polarity. Whereas floridanine showed a relatively high TPSA value of 139.67 Å^2^. These results demonstrate their ability to cross biological membranes, with extremely low or high TPSA values potentially limiting efficient membrane transport. The molar refractivity values of tris(2,4-di-tert-butylphenyl) phosphate (204.44 cm^3^/mol), stigmasterol (132.75 cm^3^/mol) and γ-sitosterol (133.23 cm^3^/mol) were found to exceed the commonly accepted range of 40–130 cm^3^/mol. Elevated molar refractivity is associated with increased molecular polarizability, which may contribute to reduced membrane permeability and poor oral bioavailability (Ibrahim et al., 2021). Log *S* values represent the aqueous solubility of a compound and are critical for evaluating its absorption potential. More negative Log *S* values indicate lower solubility, whereas values between −1 and −4 are generally considered favourable for optimal drug absorption and distribution (Liu et al., 2023). Among the evaluated compounds, 1,2,4-triazol-3-amine, 5-(1,3,5-trimethyl-4-pyrazolyl)amino showed high aqueous solubility with a Log *S* value of −1.88. Other compounds demonstrating good solubility included 5-(4-nitrophenyl)-1,3,4-oxadiazol-2(5H)-one, 7,9-di-tert-butyl-1-oxaspiro (4,5)deca-6,9-diene-2,8-dione, and floridanine, with Log *S* values ranging from −2.55 to −3.82. Moderately soluble compounds included 3-acetylphenanthrene, cyclopentadecane, and 2,4-di-tert-butylphenol, with Log *S* values between −4.41 and −4.75. Tris (2,4-di-tert-butylphenyl) phosphate (−12.89), stigmasterol (−7.46) and γ-sitosterol (−7.90) exhibited very low aqueous solubility. Overall, the drug-likeness of the phytocompounds is shown in the bioavailability radar (Figure 4). The pharmacokinetic properties of the selected phytocompounds were evaluated to understand their absorption, distribution, metabolism, and excretion (ADME) profiles. The GI absorption results revealed that compounds such as stigmasterol, γ-sitosterol, tris(2,4-di-tert-butylphenyl) phosphate, floridanine, and cyclopentadecane showed low predicted oral absorption. The BOILED-Egg analysis further supported these outcomes. In the BOILED-Egg plot, compounds that fall under the yellow region (yolk) are considered capable of crossing the blood–brain barrier (Daina and Zoete, 2016), whereas compounds that fall within the white region are predicted to have high passive GI absorption. Figure 5 illustrates that 3-acetylphenanthrene, 7,9-Di-tert-butyl-1-oxaspiro (4,5)deca-6,9-diene-2,8-dione, and 2,4-Di-tert-butylphenol fall within the BBB-permeable region, suggesting their potential to exert central nervous system activity. Further, floridanine and tris(2,4-di-tert-butylphenyl) phosphate were predicted to act as a P-gp substrate, indicating that they may undergo active efflux, particularly at the intestinal barrier and BBB. This efflux mechanism could reduce their intracellular concentration and therapeutic efficiency (Hoosain et al., 2015). Regarding cytochrome P450 inhibition, almost all the compounds were predicted to inhibit one or more CYP isoforms, indicating a potential risk of drug-drug interactions and possible interference with hepatic drug metabolism. However, γ-sitosterol, floridanine, 5-(4-nitrophenyl)-1,3,4-oxadiazol-2(5H)-one and 1,2,4-triazol-3-amine,5-(1,3,5-trimethyl-4-pyrazolyl)amino were predicted not to inhibit CYP enzymes, indicating a relatively safer metabolic profile. Toxicity predictions for the phytocompounds were performed by using the ProTox-3 web server and summarised in Table 5. The predicted median lethal dose (LD_50_) values ranged from 46 to 15,800 mg/kg, with the phytocompounds classified into toxicity classes 2–6 based on their predicted acute oral toxicity. Most of the phytocompounds were predicted as toxicity class 4, indicating that they may be harmful if consumed orally. These compounds were 3-acetylphenanthrene (LD_50_ = 599 mg/kg), 5-(4-nitrophenyl)-1,3,4-oxadiazol-2(5H)-one (680 mg/kg), 2,4-di-tert-butylphenol (700 mg/kg), stigmasterol and γ-sitosterol (890 mg/kg), 7,9-di-tert-butyl-1-oxaspiro (4,5)deca-6,9-diene-2,8-dione (900 mg/kg), and 1,2,4-triazol-3-amine, 5-(1,3,5-trimethyl-4-pyrazolyl)amino (1,000 mg/kg). Among these phytocompounds, floridanine showed the lowest predicted LD_50_ value of 46 mg/kg and was classified into toxicity class 2, indicating that it may be fatal if ingested. Cyclopentadecane was classified into toxicity class 3, with a predicted LD_50_ of 750 mg/kg, indicating higher predicted oral toxicity. Tris (2,4-di-tert-butylphenyl) phosphate was predicted to be 15,800 mg/kg and classified as class 6, indicating comparatively low predicted acute oral toxicity according to the ProTox-3 classification. Based on the SwissADME evaluation of physicochemical properties, pharmacokinetic behaviour, BOILED-Egg analysis, metabolic interaction profile, and drug-likeness parameters, together with the ProTox-3 acute oral toxicity prediction, 5-(4-nitrophenyl)-1,3,4-oxadiazol-2(5H)-one and 1,2,4-triazol-3-amine, 5-(1,3,5-trimethyl-4-pyrazolyl)amino (Figure 6) exhibited a comparatively balanced overall profile among the top ten docked phytocompounds. Both compounds showed no violations of Lipinski’s rule of five, favourable aqueous solubility and gastrointestinal absorption, and no predicted inhibition of the evaluated CYP enzymes, while their predicted acute oral toxicity was classified as class 4. Considering these combined characteristics, these two compounds were selected for further molecular dynamics simulation to evaluate the stability and dynamic behaviour of their respective protein-ligand complexes.

**TABLE 5:** SwissADME properties and acute oral toxicity profiles of the top ten docked phyto-compounds from WSPH extract.

Compound name	GI absorption	BBB permeant	P-gp substrate	CYP1A2 inhibitor	CYP2C19 inhibitor	CYP2C9 inhibitor	CYP2D6 inhibitor	CYP3A4 inhibitor	Lipinski violations	TPSA (Å^2^)	MR	Consensus Log *P*	Log *S*	LD50 mg/kg	Toxicity class
Stigmasterol	Low	No	No	No	No	Yes	No	No	1	20.23	132.75	6.98	−7.46	890	4
γ-sitosterol	Low	No	No	No	No	No	No	No	1	20.23	133.23	7.24	−7.9	890	4
3-Acetylphenanthrene	High	Yes	No	Yes	Yes	No	No	No	0	17.07	71.65	3.77	−4.41	599	4
Tris (2,4-di-tert-butylphenyl) phosphate	Low	No	Yes	No	No	Yes	No	Yes	2	54.57	204.44	12.15	−12.89	15,800	6
Floridanine	Low	No	Yes	No	No	No	No	No	0	139.67	111.62	0.51	−2.86	46	2
Cyclopentadecane	Low	No	No	No	No	Yes	No	No	1	0	72.1	5.1	−4.75	750	3
7,9-Di-tert-butyl-1 oxaspiro (4,5)deca-6,9-diene-2,8-dione	High	Yes	No	No	Yes	Yes	No	No	0	43.37	79.66	3.4	−3.82	900	4
5-(4-nitrophenyl)-1,3,4-oxadiazol-2(5H)-one	High	No	No	No	No	No	No	No	0	96.84	56.1	1.17	−2.55	680	4
1,2,4-triazol-3-amine, 5-(1,3,5-trimethyl-4-pyrazolyl)amino	High	No	No	No	No	No	No	No	0	97.44	57.31	0.37	−1.88	1,000	4
2,4-Di-tert-butylphenol	High	Yes	No	No	No	No	Yes	No	0	20.23	67.01	3.99	−4.55	700	4

**FIGURE 4:**
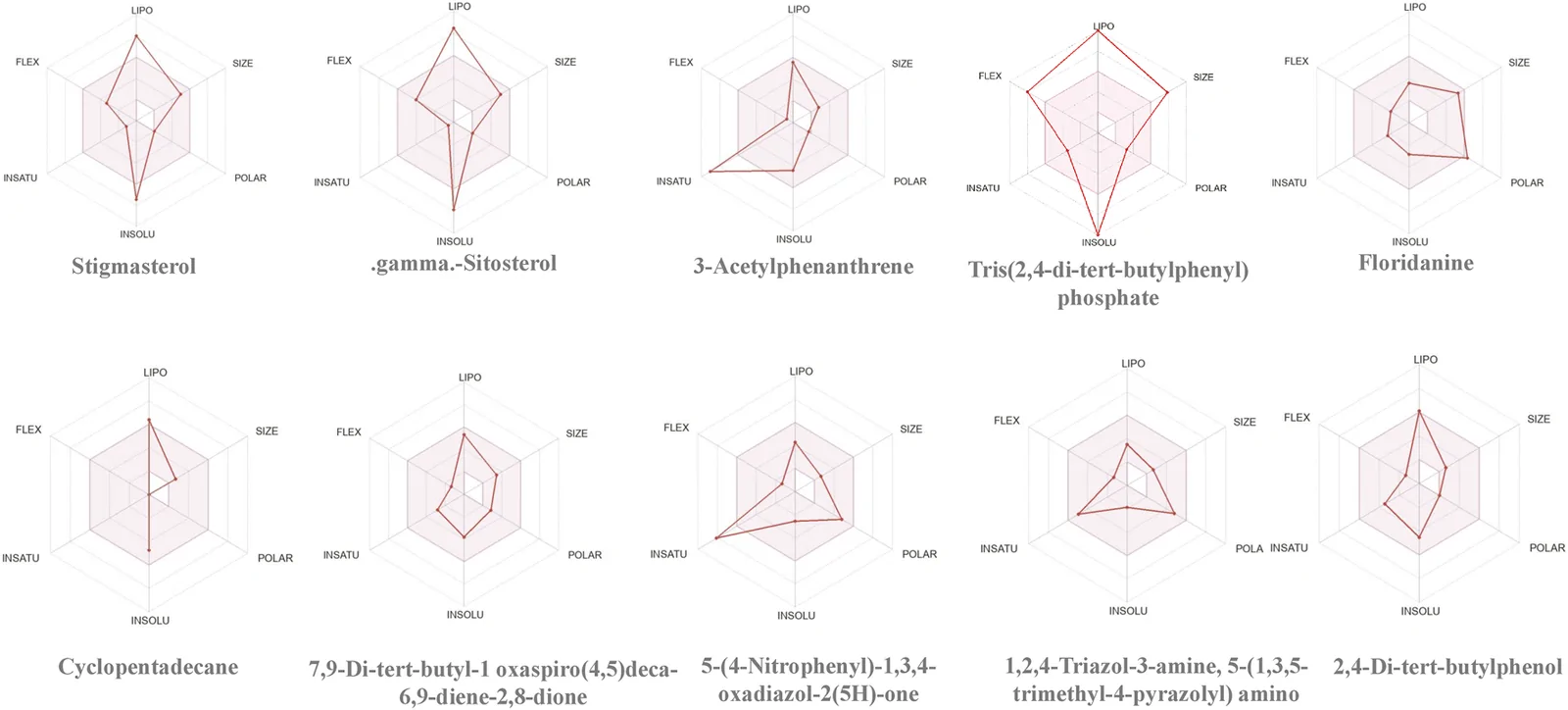
Bioavailability radar of the top ten docked phytocompounds from WSPH extracts.

**FIGURE 5:**
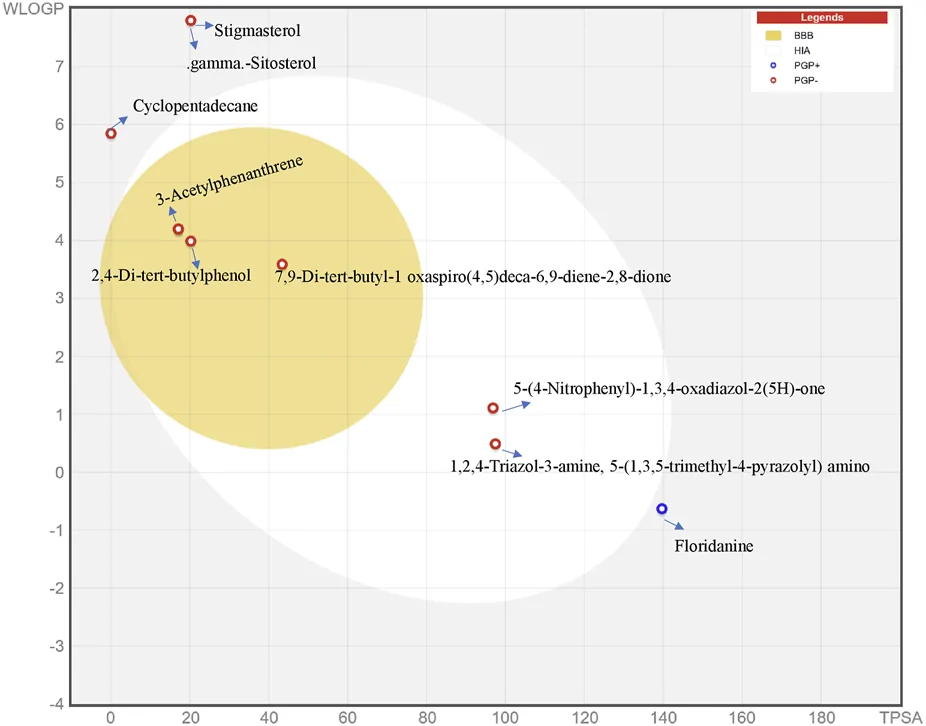
Graphical representation of the BOILED-Egg analysis of the top ten docked phytocompounds from WSPH extracts.

**FIGURE 6:**
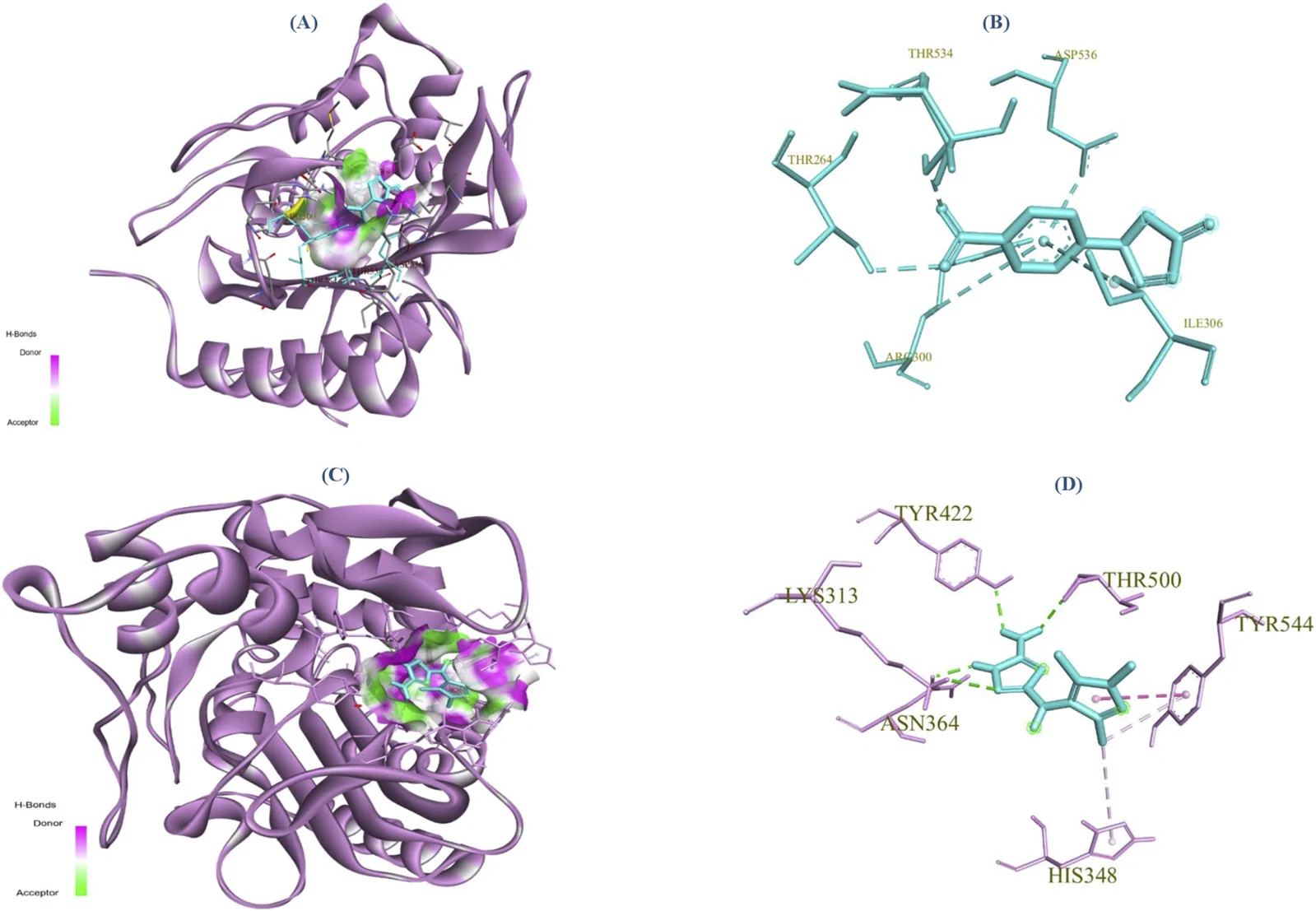
**(A,B)** Three-dimensional interaction structures of 5-(4-nitrophenyl)-1,3,4-oxadiazol-2(5H)-one with PBP2; **(C,D)** three-dimensional interaction structures of 1,2,4-triazol-3-amine, 5-(1,3,5-trimethyl-4-pyrazolyl)amino with PBP2.

### 3.4 Molecular dynamics simulation

Based upon the outcomes of the ADME and molecular docking studies, we considered two ligand [5-(4-nitrophenyl)-1,3,4-oxadiazol-2(5H)-one (compound 1) and 1,2,4-triazol-3-amine, 5-(1,3,5-trimethyl-4-pyrazolyl)amino (compound 2)] complexes for the molecular dynamics simulation analysis in comparison with the apo form of the protein target (PBP2) over a time frame of 100 ns. To evaluate ligand-complex structural stability, RMSD of the backbone atoms was calculated across the simulation trajectory by measuring the deviations of the complexes relative to the apo structure (Javali and Thirumurugan, 2024). The RMSD profiles (Figure 7) illustrate the comparative stability of the apo protein and the ligand-bound complexes. The results indicate that the compound 1 complex (∼0.09 nm–∼0.19 nm) exhibits greater stability compared to the apo protein (∼0.09 nm–∼0.24 nm) and the compound 2 complex (∼0.08 nm–∼0.23 nm), while the control_PBP2 complex maintained a stable RMSD trend, ranging from ∼0.09 to ∼0.18 nm, with an average RMSD across 100 ns 0.1365 nm (Supplementary Figure S2). Initially, the apo form of PBP2 equilibrated from 0 to ∼45 ns. After this, the apo protein showed increased deviation during the latter part of the simulation, with an average RMSD of 0.151 nm. Whereas the compound 1_PBP2 complex maintained reduced structural divergence. The RMSD plot remained stable throughout the simulation, with an average of 0.139 nm, indicating enhanced stabilisation of the protein upon ligand binding. The compound 2_PBP2 complex has the highest mean RMSD value (0.158 nm) compared to the apo protein. This increased deviation is attributable to initial structural fluctuations observed from 0 to ∼50 ns. After 50 ns, the system reached equilibration and subsequently stabilized throughout the simulation. The RMSF of the Cα atoms for each residue was computed (Chauhan and Sharma, 2025) to analyse atomic fluctuations of the residues and is presented in Figure 7. The RMSF plot suggests that residue fluctuations were comparatively reduced upon ligand binding compared with the apo protein. Overall, most residue fluctuations ranged from ∼0.04 nm to ∼0.15 nm. Furthermore, the mean residue fluctuation was reduced compared to the apo protein (apo: ∼0.088 nm; compound 1_PBP2 complex: ∼0.080 nm; compound 2_PBP2 complex: ∼0.081 nm), with the control_PBP2 complex showing a mean RMSF of 0.0759 nm (Supplementary Figure S3). This reduced residue fluctuations suggests enhanced structural stability upon complex formation. In order to understand the compactness of protein before and after binding of ligand we performed Rg analysis (Ghahremanian et al., 2022), and the values of apo protein and ligand complexes were compared. Figure 7 shows the Rg values of apo and complex forms ranging from ∼1.88 nm to ∼1.94 nm. Then, the mean radius of gyration (Rg) of the apo protein was 1.896 nm, compared with 1.906 nm for the compound 1_PBP2 complex and 1.905 nm for the compound 2_PBP2 complex. The control_PBP2 complex showed a mean Rg value of 1.927 nm (Supplementary Figure S4). The observed Rg values reflects the overall compactness of the protein structure and indicating that the structural compactness of PBP2 was broadly maintained across the simulated systems. SASA evaluates the extent of solvent exposure of the protein surface and reflects changes in compactness and solvent accessibility associated with ligand binding (Durham et al., 2009). Figure 7 shows that SASA of the complexes was observed to be higher than that of the apo protein throughout the simulation trajectory. Although the complex shows slightly higher SASA compared to the apo structure, the RMSD and Rg analyses confirm that the protein remains structurally stable. The increase in SASA likely results from ligand-induced conformational adjustments in the binding pocket that expose additional residues to the solvent, which is commonly observed in protein–ligand interactions (Elayyan et al., 2025).

**FIGURE 7:**
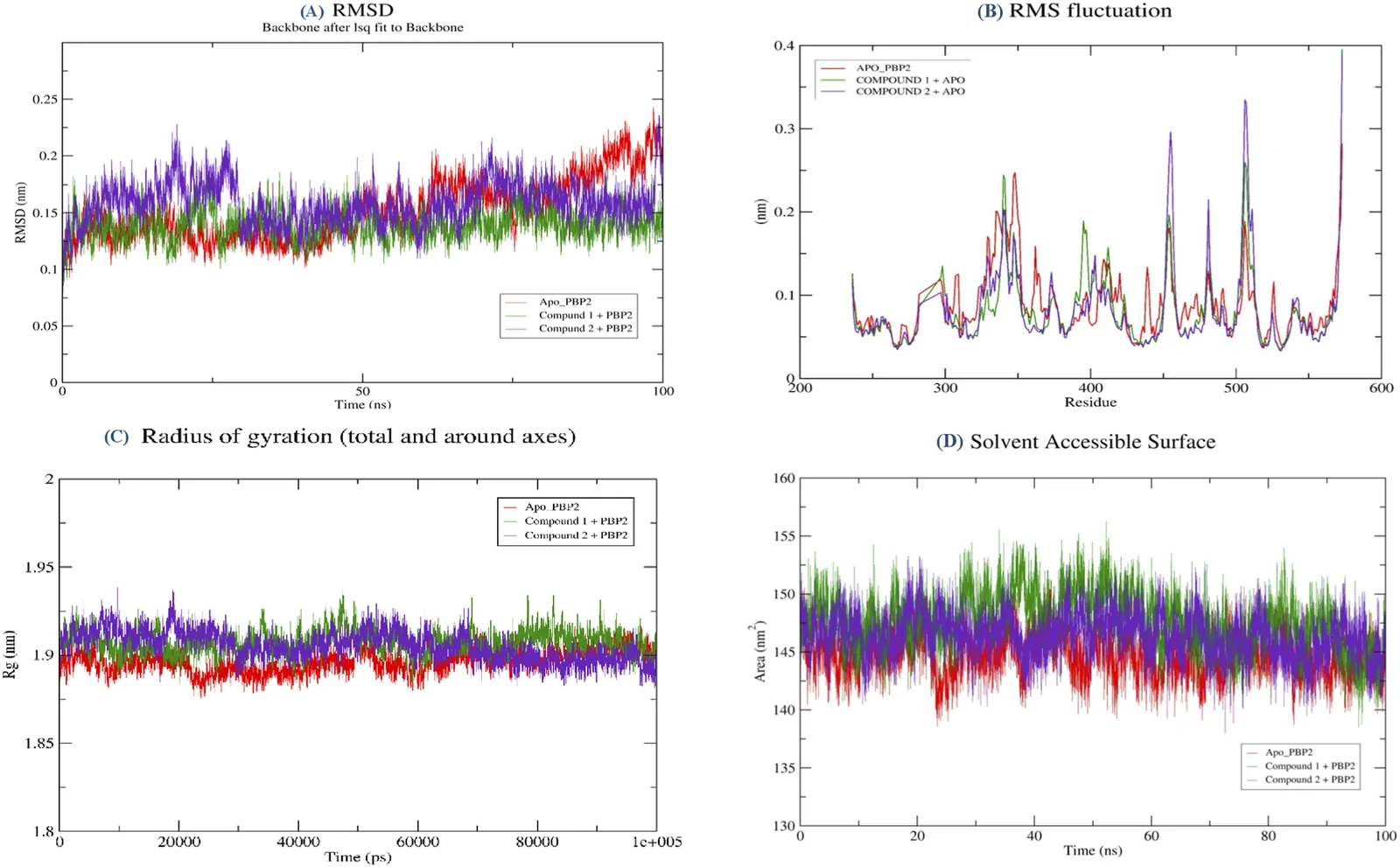
Molecular dynamics simulation analysis of Apo_PBP2, Compound 1_PBP2, Compound 2_PBP2 **(A)** RMSD **(B)** RMSF **(C)** Rg **(D)** SASA.

Hydrogen bond analysis over the simulation period revealed persistent intermolecular interactions for both complexes (Figure 8). Throughout the simulation, the compound 1_PBP2 complex predominantly maintained 1–3 hydrogen bonds with occasional transient increases up to 4–5 bonds, indicating stable and sustained binding. Compound 2_PBP2 complex exhibited a comparable hydrogen-bonding interaction, largely fluctuating between 1 and 3 bonds with brief higher-order interactions. Overall, both complexes maintained consistent hydrogen bond occupancy, supporting stable ligand accommodation within the PBP2 binding pocket.

**FIGURE 8:**
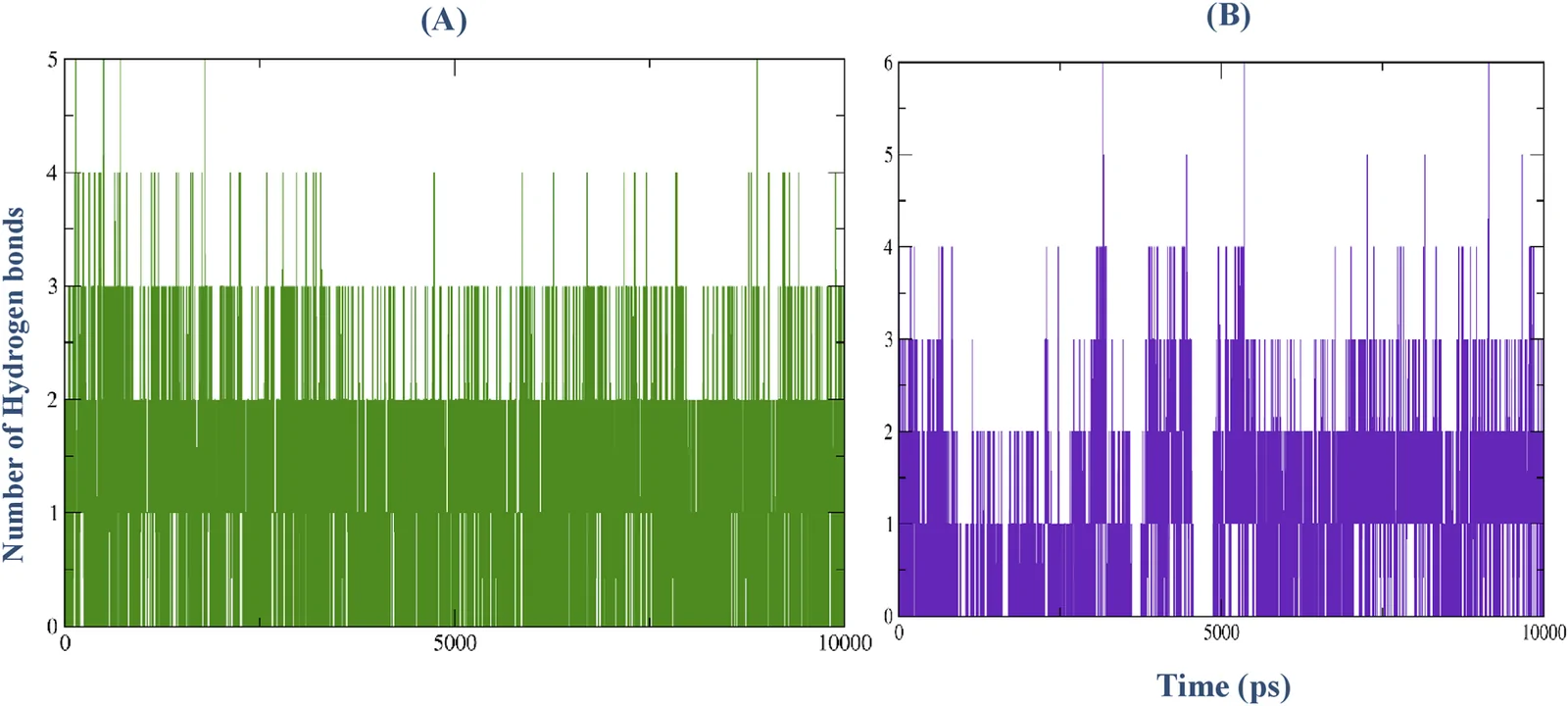
Intermolecular hydrogen bonding analysis **(A)** Compound 1_PBP2 **(B)** Compound 2_PBP2.

### 3.5 PCA and FEL analysis

In this study, PCA was utilized to delineate the collective motions of PBP2 across the apo and ligand-bound states and to evaluate whether binding the ligands altered its conformational landscape during the MD simulation. PCA was performed using the eigenvectors of the covariance matrix computed from MD trajectories. PCA enables the reduction of complex, high-dimensional atomic motion into a few principal components (PCs) by calculating the eigenvectors and eigenvalues of the covariance matrix derived from the positional fluctuations of the Cα atoms over the trajectory (Joshi et al., 2022; Elayyan et al., 2025). These principal components correspond to collective motions, which are often functionally relevant. The 2D projection of the first two principal components (PC1 and PC2) was illustrated in Figure 9. The apo structure of PBP2 exhibits a broader conformational space, indicating enhanced intrinsic flexibility and dynamic heterogeneity. The illustrated 2D projection shows that upon compounds binding the conformational landscape of the PBP2 complex was markedly altered. The compound 1 complex displays a more compact and centralized clustering pattern relative to the apo PBP2, reflecting restricted large-scale motions and reduced conformational sampling. Whereas the compound 2 complex, although exhibiting some degree of confinement compared to the apo_PBP2, demonstrates comparatively wider dispersion, particularly along PC1, suggesting residual flexibility. Collectively, the reduced phase-space dispersion observed for both ligand-bound systems relative to the apo form underscores ligand-induced dynamic stabilization of PBP2, with compound 1 exerting a more prominent restricted effect on the global conformational space.

**FIGURE 9:**
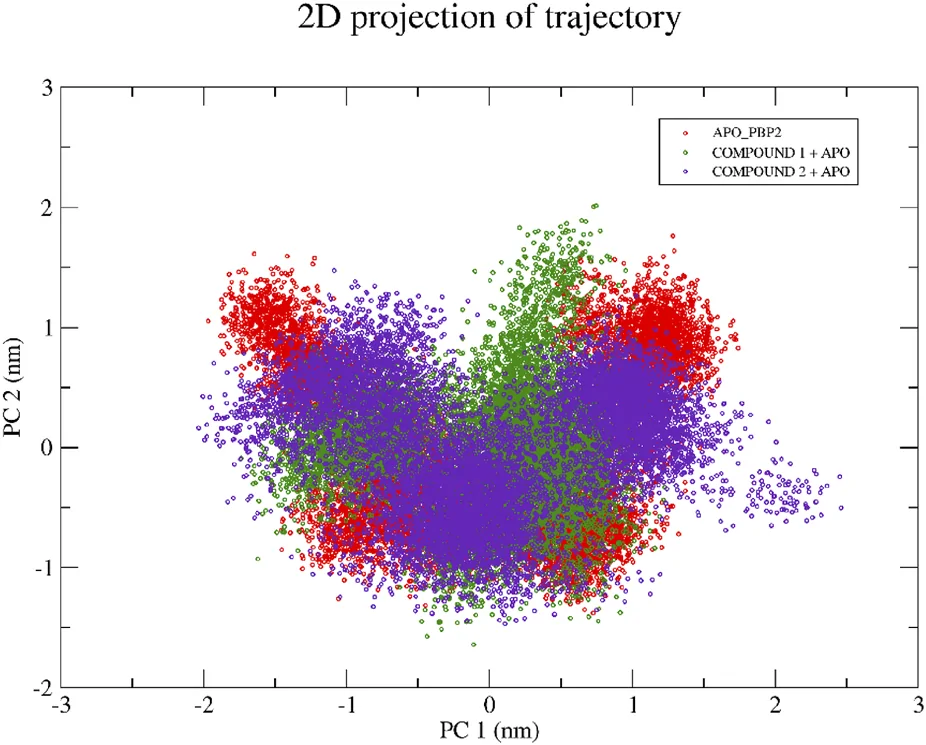
2D projection of principal component analysis showing the distribution of conformations along the first two eigenvectors (PC1 and PC2) for the apo protein and ligand bound complexes.

Furthermore, FEL analysis on the PCA trajectories was carried out to investigate low energy conformations in the test systems involving the protein folding of apo_PBP2, compound 1_PBP2 complex and compound 2_PBP2 complex. FEL analysis was used to determine the most thermodynamically stable conformation of protein-ligand complex (0 kcal/mol) from conformational ensemble generated during MD simulation. Two variables (PC1 and PC2), each reflecting specific system properties, were utilized to capture system variability in order to represent this landscape (Alkhathami et al., 2025). The FEL plots were shown in Figure 10, allowing us to examine the global minima of these systems, where the dark blue regions indicate the conformational states with the lowest free energy. The free energy landscape of the apo_PBP2 shows several separate low-energy regions spread widely across PC1 and PC2, indicating that the protein adopts multiple conformations in the absence of a ligand. Upon binding of compound 1, the energy surface became more concentrated with a single prominent deep minimum, suggesting that the protein settles into a more stable and well-defined structure compared to apo_PBP2. However, the compound 2_PBP2 complex still displays more than one accessible low-energy region and a comparatively wider distribution of energy landscape than compound 1_PBP2. This indicates that although compound 2 stabilizes the protein relative to the apo PBP2, it allows slightly more structural movement than compound 1. Overall, both ligands reduce conformational variability compared to the apo form of protein, with compound 1 providing the strongest stabilization effect.

**FIGURE 10:**
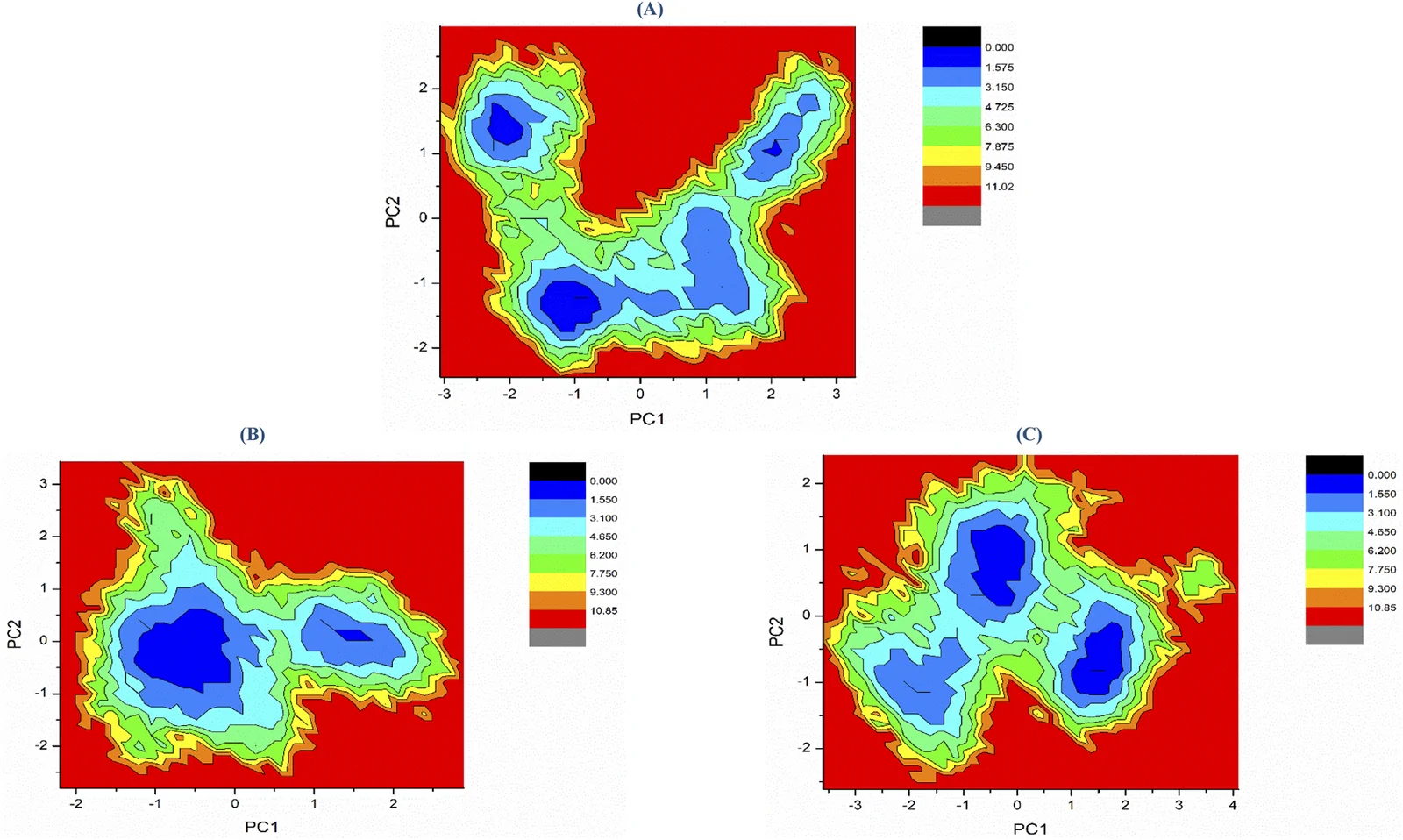
2D plots of Free energy landscapes **(A)** Apo_PBP2 **(B)** compound 1_PBP2 **(C)** Compound 2_PBP2.

### 3.6 Binding free energy calculation

The MM-PBSA method was used to calculate the binding free energy and evaluate the interaction potential of PBP2 with Compound 1 and Compound 2. This approach estimates the thermodynamic favourability of ligand-protein interactions. The contributions from electrostatic (ΔEEL) and Van der Waals interactions (ΔVDWAALS), polar solvation energy (ΔEGB), non-polar solvation energy (ΔESURF), and SASA-related energies were combined to calculate the total binding free energy (ΔTOTAL) (Wang et al., 2019; Elayyan et al., 2025). The average binding free energy and standard deviations for each compound are summarized in Table 6. Compounds 1 and 2 have binding free energies of −19.11 ± 2.29 kJ/mol and −11.16 ± 2.34 kJ/mol, respectively. These values indicate that both compounds have favourable interactions with PBP2. Substantial Van der Waals and electrostatic interactions were observed for both ligands, contributing significantly to the overall stability of complexes. Comparatively, Compound 1 demonstrated a more favourable binding free energy than Compound 2. These MM-PBSA results are consistent with the earlier dynamic analyses, further supporting that both compounds can form stable and energetically favourable interactions with PBP2.

**TABLE 6:** Binding free energy calculations using the MM-PBSA tool for Compound 1_PBP2 and Compound 2_PBP2.

Complex	ΔVDWAALS (kJ/mol)	ΔE_EL_ (kJ/mol)	ΔE_GB_ (kJ/mol)	ΔE_SURF_ (kJ/mol)	ΔG_GAS_ (kJ/mol)	ΔG_SOLV_ (kJ/mol)	ΔTOTAL (kJ/mol)
Compound 1_PBP2	−26.60 ± 2.28	−30.17 ± 4.57	41.74 ± 3.52	−4.08 ± 0.13	−56.77 ± 4.61	37.66 ± 3.48	−19.11 ± 2.29
Compound 2_PBP2	−18.11 ± 2.09	−16.51 ± 4.64	25.24 ± 3.78	−1.78 ± 0.20	−34.62 ± 5.35	23.46 ± 3.74	−11.16 ± 2.34

## 4 Conclusion

Antimicrobial resistance in *Neisseria gonorrhoeae* continues to pose a serious challenge to current treatment strategies, particularly with the emergence of highly drug-resistant strains like H041, which has created a critical need for the discovery of reliable therapeutic agents. Targeting essential proteins involved in bacterial cell wall synthesis has therefore become an important approach for identifying new therapeutic candidates. In this study, phytocompounds derived from the *Borassus flabellifer* L. haustorium extract were investigated as potential inhibitors of PBP2 using a systematic computational framework. Ultrasound-assisted extraction with different solvent polarities was utilized to obtain a wide range of phytochemicals. The compounds identified from these extracts were further evaluated for their interaction with the PBP2 protein, followed by ADMET evaluation to assess their pharmacokinetic suitability. Based on these analyses, 5-(4-nitrophenyl)-1,3,4-oxadiazol-2(5H)-one and 1,2,4-triazol-3-amine,5-(1,3,5-trimethyl-4-pyrazolyl)amino, were identified as promising compounds for further analysis. Analysis of RMSD, RMSF, Rg, SASA, and hydrogen bond formation indicated that both ligands have stable interactions within the binding pocket of PBP2 throughout the 100 ns simulation period. Furthermore, MM-PBSA for binding free energy calculations supported the favourable interactions of the selected compounds with the target protein (PBP2). Additionally, PCA and FEL analysis revealed stable conformational states of the complexes during the simulation trajectory. During the simulation, 5-(4-nitrophenyl)-1,3,4-oxadiazol-2(5H)-one maintained greater binding stability with PBP2 compared to 1,2,4-triazol-3-amine, 5-(1,3,5-trimethyl-4-pyrazolyl)amino, indicating its potential as a promising candidate for further investigation against drug-resistant *N. gonorrhoeae*. Overall, the findings of this study suggest that phytochemicals derived from the WSPH extract, particularly 5-(4-nitrophenyl)-1,3,4-oxadiazol-2(5H)-one, may serve as promising lead molecules for the development of new therapeutic strategies against drug-resistant gonococcal infections. These findings provide a computational base for further experimental validation to develop new therapeutics against resistant gonococcal infections. Nevertheless, further *in vitro* and *in vivo* experimental validation will be necessary to validate the antimicrobial efficacy and therapeutic applicability of the identified compound.

## 5 Limitations and future scope

The present study is limited to *in silico* evaluation of the identified phytocompounds. Experimental validation, including MIC determination, antimicrobial assays, and target-specific studies against resistant *N. gonorrhoeae* strains, remains necessary.

## Data Availability

The original contributions presented in this study are included in the article and Supplementary Material. Further inquiries can be directed to the corresponding author.
