# Supplementary material for: In silico evaluation of potential penicillin-binding protein 2 (PBP2) inhibitors from Borassus flabellifer haustorium against cephalosporin-resistant Neisseria gonorrhoeae
Source: Front Pharmacol. 2026 Oct 9;17:1918221. doi: 10.3389/fphar.2026.1918221 (PMC13690551; doi:10.3389/fphar.2026.1918221)
Supplement: Supplementary file 1 [file DataSheet1.docx]

**Supplementary material**

Figure S1: (A) Redocking validation of the co-crystallized ligand with PBP2. (B) 2D molecular interaction diagram of ceftriaxone with PBP2.

Figure S2: RMSD analysis of the control_PBP2 complex during the molecular dynamics simulation.

Figure S3: RMSF analysis of the control_PBP2 complex during the molecular dynamics simulation.

Figure S4: Radius of gyration (Rg) analysis of the control _PBP2 complex during the molecular dynamics simulation.

Figure S5: Solvent-accessible surface area (SASA) analysis of the control_PBP2 complex during the molecular dynamics simulation.

Figure S6: Intramolecular hydrogen bond analysis of the control_PBP2 complex during the molecular dynamics simulation.
