# Supplementary material for: In silico evaluation of potential penicillin-binding protein 2 (PBP2) inhibitors from Borassus flabellifer haustorium against cephalosporin-resistant Neisseria gonorrhoeae
Source: Front Pharmacol. 2026 Oct 9;17:1918221. doi: 10.3389/fphar.2026.1918221 (PMC13690551; doi:10.3389/fphar.2026.1918221)
Supplement: Supplementary file 2 [file DataSheet2.docx]

**In Silico Evaluation of Potential Penicillin-Binding Protein 2 (PBP2) Inhibitors from *Borassus flabellifer* Haustorium Against Cephalosporin-Resistant *Neisseria gonorrhoeae***

*Udhaya Rajasekaran^1^, T. B. Sridharan^1*^*

^1^Department of Integrative Biology, School of Biosciences and Technology, Vellore Institute of Technology, Vellore, Tamil Nadu, India - 632014.

*Corresponding author*:* Dr. T. B. Sridharan

E-Mail : [tbsridharan@vit.ac.in](mailto:tbsridharan@vit.ac.in)

Figure S2.1. Mass spectra of constituents identified in the methanol extract of WSPH by GC–MS (peaks 1–29).

Figure S2.2. Mass spectra of constituents identified in the ethyl acetate extract of WSPH by GC–MS (peaks 1–28).

Figure S2.3. Mass spectra of constituents identified in the hexane extract of WSPH by GC–MS (peaks 1–12).
